# RESONANCE: Protocol for a Nationwide Cohort Study of Chronic Liver Diseases Using the French National Health Data System

**DOI:** 10.2196/89577

**Published:** 2026-10-05

**Authors:** Aurore Baron, Laure Tron, Frédéric Balusson, Vaele Zannou, Victoria Mignot, Anne Ego, Anna Borowick, Thomas Decaens, Emmanuel Oger, Sébastien Bailly, Charlotte Costentin

**Affiliations:** 1 Université Grenoble Alpes Institute for Advanced Biosciences, Research Center UGA Inserm U 1209 - CNRS UMR 5309, Team Cell Dynamics, Metabolism & Cancer (DIMAC) Grenoble, Île-de-France France; 2 Université Paris Saclay Centre Hépato-Biliaire, Hôpital Paul-Brousse, Inserm UMR 1193 Villejuif France; 3 Université Grenoble Alpes Hepato-Gastroenterology and Digestive Oncology Department, CHU Grenoble Alpes La Tronche, Grenoble France; 4 Pharmacovigilance and Pharmacoepidemiology, CHU Rennes Rennes France; 5 Université Rennes CHU Rennes, Inserm, EHESP, Irset, UMR_S 1085 Rennes France; 6 Université Grenoble Alpes Pôle Santé Publique, Centre Hospitalier Universitaire de Grenoble Grenoble France; 7 Université Grenoble Alpes TIMC-IMAG, UMR 5525, CNRS, Equipe ThEMAS Grenoble France; 8 Université Grenoble Alpes HP2 Laboratory, Inserm U1300 Grenoble France

**Keywords:** population-based cohort, French National Health Data System, SNDS, chronic liver diseases, health care trajectories, administrative health data, epidemiology, real-world data

## Abstract

**Background:**

Chronic liver diseases (CLDs) are frequent in Europe, including in France, and are mainly driven by alcohol, metabolic dysfunction (diabetes and obesity), and viral hepatitis. Although risk factors are well established and easy to identify, CLDs are frequently diagnosed at an advanced stage, translating into poor prognosis. Descriptive data on CLD burden in France remain scarce, and health trajectories of these patients are uncharted. However, such data are crucial to guide clinical practice, to determine the target population for personalized public health policies, and to develop innovative strategies to reduce inequities in access to health care and ultimately improve survival.

**Objective:**

The aim of the French RESONANCE cohort is to provide a detailed description of CLD burden in France and study health trajectories.

**Methods:**

The RESONANCE cohort was derived from the French National Health Data System (Système National des Données de Santé [SNDS]). Patients with at least one CLD-specific *ICD-10* (*International Statistical Classification of Diseases, Tenth Revision*) code (including primary liver cancer [PLC] and rare diseases), or related procedure, biology, or drug and/or a cause of death related to one of these *ICD-10* codes, were targeted from the 2% representative SNDS sample (ESND [Échantillon du Système National des Données], Simplified Sample of the French National Health Data System). Demographic characteristics, risk factors, comorbidities, etiologies, social environment and health care accessibility, as well as severity of the liver disease at first identification and medical management, were assessed. This paper describes the procedures used to build the cohort, outlines main variables, and defines the research objectives.

**Results:**

Overall, 26,663 incident and prevalent CLD cases identified between 2015 and 2021 were included in the RESONANCE cohort. Men accounted for 58.1% (15,490) of the cohort, and the mean age was 59.9 (SD 17.4) years. The cohort covers a broad spectrum of etiologies, including alcohol-related liver disease (7580/26,663, 28.4%), metabolic-associated liver disease (6030/26,663, 24.8%), viral hepatitis (3450/26,663, 12.9%), and rare liver diseases (839/26,663, 3.1%). A total of 43% (11,461/26,663) of the patients lived in a socially deprived environment (according to the 4th and 5th quintiles of the French Deprivation Index).

**Conclusions:**

This paper will enable a detailed, real-world analysis of epidemiology, care trajectories, and outcomes of CLDs across France. With a particular focus on gender and socioeconomic disparities, it offers a unique opportunity to identify vulnerable populations and inform proportionate universalism strategies in prevention and care.

**International Registered Report Identifier (IRRID):**

DERR1-10.2196/89577

## Introduction

### Background

Chronic liver disease (CLD), culminating in cirrhosis and primary liver cancer (PLC), is highly prevalent, affecting approximately 1.5 billion individuals worldwide [1-3]. The main etiologies are well established and include alcohol-related liver disease (ALD), metabolic dysfunction–associated steatotic liver disease (MASLD), and chronic viral infections caused by hepatitis B virus (HBV) and hepatitis C virus (HCV). Although viral infections are better controlled in developed countries due to HBV vaccination and effective antiviral therapies, alcohol misuse remains widespread, and the incidence of metabolic syndrome is rising, leading to a global increase in CLD prevalence, particularly MASLD. In Europe, the estimated prevalence of CLD is 833 cases per 100,000 inhabitants (range 447-1100), with an estimated cirrhosis incidence of 26.0 per 100,000 inhabitants [4,5]. Altogether, CLD remains associated with poor outcomes, accounting for approximately 2 million deaths annually worldwide [6].

In France, data on the epidemiology of CLD and its natural history are limited, and previous national studies predominantly focused on PLC (especially hepatocellular carcinoma [HCC]) and MASLD [7,8] through cancer registries [9,10] (lacking clinical data) or cohort studies (lacking representativeness) [11,12]. In the French Network of Cancer Registries (Francim), 11,658 new cases of PLC were declared in 2023 (8874 in men and 2784 in women) with a 12.3 standardized incidence rate for men and 3.1/100,000 for women [13]. Mortality related to CLD is poorly documented; however, it is estimated that cirrhosis causes approximately 15,000 deaths annually [14], with PLC responsible for 8700 deaths in 2018, accompanied by a dismal 5-year standardized net survival rate of 18% according to Institut National du Cancer in 2023 [10,15]. Cirrhosis is most often identified during a severe complication such as variceal bleeding, ascites, liver failure, or PLC [16-20]. Furthermore, more than half of PLC cases are diagnosed at advanced stages, precluding curative treatment options, particularly in patients with ALD [21-24]. This delayed diagnosis is related both to underrecognition of the underlying liver disease and to suboptimal implementation of HCC surveillance. Although international guidelines recommend semiannual surveillance using liver ultrasound with or without alpha-fetoprotein (AFP) in case of cirrhosis [25-27], real-world surveillance rates remain critically low, with only approximately 20%-25% of eligible patients undergoing appropriate surveillance [28]. Delayed diagnosis of advanced fibrosis or PLC markedly worsens patient prognosis.

Early medical intervention in at-risk populations is essential for preventing CLD onset. Such interventions include managing obesity, treating type 2 diabetes, screening for alcohol misuse, and providing addiction care. Even after CLD has developed, controlling the underlying causes can slow disease progression to advanced fibrosis stages and/or PLC, thereby reducing liver-related and overall mortality [11,29-32]. Since advanced fibrosis is the strongest predictor of clinical outcomes and mortality, early identification of significant fibrosis is critical, especially in high-risk groups such as individuals with alcohol misuse, diabetes, obesity, or sleep apnea syndrome. Several noninvasive tests have been developed to identify patients at risk of advanced fibrosis [33], with the European Association for the Study of the Liver (EASL) recommending the use of the FIB-4 score in high-risk populations [34].

Despite well-known etiologies and new tools for early fibrosis identification, CLD is often diagnosed at late stages of fibrosis or at the onset of PLC, indicating gaps in health care pathways.

A better understanding of CLD epidemiology, natural history, and recent changes (increase in MASLD, for instance [35], or a new therapeutic era for PLC with the advent of immunotherapy [36-38]), and health care trajectories is crucial to identify factors driving its poor prognosis [39].

To address this knowledge gap, we established a cohort of patients with CLD leveraging data from the French National Health Data System (Système National des Données de Santé [SNDS]). This paper describes the procedures used to target the population to be included in the cohort, construct the variables for the analyses, and define the research objectives.

### Objectives

RESONANCE is an historical cohort derived from the SNDS, including both incident and prevalent cases of CLD from 2015 to 2021.

The primary research objective is to assess the impact of nonoptimal care pathways on the incidence of severe forms of CLD. Secondary objectives include the following:

1. estimate the incidence, prevalence, and mortality rates from CLD from 2015 to 2021,
2. evaluate the quality of monitoring and management of CLD from 2015 to 2023 (with a focus on the COVID-19 pandemic period),
3. identify sociodemographic, medical, geographic, and temporal factors likely to influence the occurrence of severe forms of CLD, and
4. analyze the outcome (occurrence of complications and survival) of patients with CLD (overall and stratified according to each type of CLD) by extending the follow-up of the cohort until December 31, 2023.

We plan to perform analyses dedicated to subpopulations of special interest such as patients with cirrhosis, PLC, and subtypes of PLC. In particular, we aim to describe the therapeutic management of HCC (first-line and subsequent strategies) and changes over time, especially in the immunotherapy era (since 2021).

For each objective, we will explore the role of sex, age, social environment, and access to care, as well as the COVID-19 pandemic period.

## Methods

### Data Sources

#### SNDS

The SNDS is a comprehensive repository of pseudonymized medico-administrative data, covering approximately 98%-99% of the 68 million people in France and encompassing all health care services reimbursed by the national health system [40]. The SNDS enables the integration of data from multiple sources, including health insurance (SNIIRAM [Système national d'information inter-régimes de l'Assurance maladie]) that comprises the individual health care consumption data (Données de Consommation Inter-Régimes [DCIR]), hospitalizations (Programme de médicalisation des systèmes d'information [PMSI]), and medical causes of death produced by the Center for Epidemiology of Medical Causes of Death (Centre d'épidémiologie sur les causes médicales de décès [CépiDc]).

#### ESND: SNDS 2% Sample

A 2% representative (according to sex, age, major insurance schemes, region, and French department of residence of the beneficiary) sample of the SNDS (known as ESND [Échantillon du Système National des Données]) is now available. Using a large representative sample instead of the full SNDS ensures feasibility and methodological robustness while avoiding the technical, computational, and environmental burden of full SNDS extraction, which is both time-consuming and costly. The RESONANCE cohort was extracted from the ESND dataset. This approach is consistent with current French regulatory and institutional recommendations promoting data minimization and responsible use of large-scale health data infrastructures.

### Regulatory Procedures

Permanent access to the SNDS is automatically granted to certain government agencies and public institutions, such as university-affiliated hospitals and public service authorities. The scope of data access is defined by a Council of State decree, based on the opinion of the National Commission for Information Technology and Civil Liberties (CNIL [French Data Protection Authority]). The RESONANCE cohort is promoted by Grenoble Alpes University Hospital, which holds permanent access to SNDS data, exempting it from additional regulatory procedures [41]. All necessary accreditations for secure data access have been obtained and RESONANCE is registered in the Health Data Hub under reference number F20230224144925 (project number 26266080). According to French regulation (Articles L1461-1 and R1461-13 of the French Public Health Code), studies conducted using pseudonymized SNDS data do not require approval from an institutional review board (IRB) or ethics committee, or patient individual consent, since no directly identifiable data are used and no contact with patients occurs.

### Cases Identification

#### Overview

To target patients to be included in the cohort, a multisource algorithmic strategy was implemented to maximize sensitivity and capture complementary clinical situations.

The RESONANCE cohort includes all the beneficiaries present in the ESND and identified with a CLD between 2013 and 2021, defined by at least 1 of the 4 possible conditions as described below and illustrated in Figure 1.

**Figure 1:**
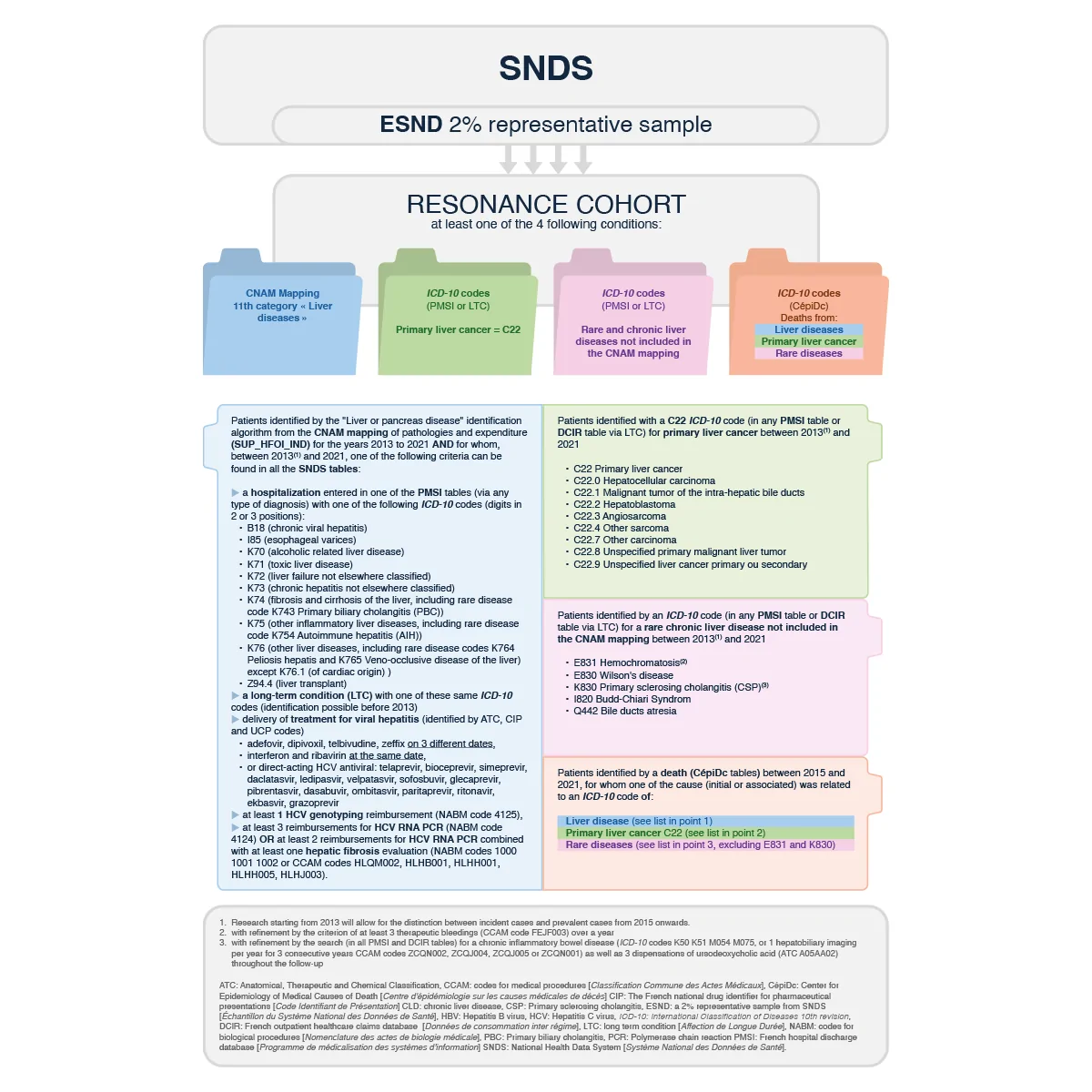
Algorithm for targeting the population included in the RESONANCE cohort. A high-resolution version of this figure is available in Multimedia Appendix 1. CNAM: Caisse Nationale d’Assurance Maladie (National Health Insurance Fund); ESND: Échantillon du Système National des Données (Simplified Sample of the French National Health Data System); ICD-10: International Statistical Classification of Diseases, Tenth Revision; LTC: long-term condition; PMSI: Programme de médicalisation des systèmes d'information; SNDS: Système National des Données de Santé (French National Health Data System).

#### Main CLD Cases

The French National Health Insurance Fund (Caisse Nationale d’Assurance Maladie [CNAM]) provides a mapping with specific algorithms to identify 50 diseases and their associated expenditures within the SNDS [40,42,43]. We preselected by this algorithm all patients from the ESND identified with a “liver or pancreatic disease” (referred to as the 11th category of the mapping), and we ultimately included only the patients with at least one condition strictly corresponding to the identification of liver diseases (conditions and codes are described in Figure 1).

#### PLC Cases

PLC-associated *ICD-10* (*International Statistical Classification of Diseases, Tenth Revision*) codes are not included in the CNAM mapping for CLD, precluding the identification of patients with PLC as the first diagnosis of CLD. Therefore, the second possible inclusion condition was the presence of at least one of the following *ICD-10* codes in any of the PMSI database (recorded as main or associated diagnosis) or associated with a long-term condition (LTC [*Affection Longue Durée*], a legal status in the French health care system granting full reimbursement for care related to specific chronic or severe illnesses requiring long-term treatment) in the DCIR database: C22 for PLC, C22.0 for HCC, C22.1 for malignant tumor of the intrahepatic bile ducts, C22.2 for hepatoblastoma, C22.3 for angiosarcoma, C22.4 for other sarcoma, C22.7 for other carcinoma, C22.8 for unspecified primary malignant liver tumor, and C22.9 for primary or secondary unspecified malignant liver tumor.

#### Rare Liver Diseases Cases

Only a limited number of rare chronic liver diseases are captured within the CNAM mapping (ie, K74.3 primary biliary cholangitis [PBC], K75.4 autoimmune hepatitis [AIH], K76.4 peliosis hepatis, and K76.5 veno-occlusive disease of the liver). Identifying rare liver diseases is challenging because only a few have specific *ICD-10* codes. Therefore, the third possible inclusion condition was the presence of at least one of the following *ICD-10* codes in any of the PMSI database (recorded as main or associated diagnosis) or associated with a LTC in the DCIR database: E83.0 for Wilson disease, I82.0 for Budd-Chiari syndrome, Q44.2 for biliary atresia, K83.0 for primary sclerosing cholangitis (PSC) only if combined with associated bowel disease diagnoses or imaging, as well as ursodeoxycholic acid (UDCA) treatment as described by Corpechot et al [44] (codes are described in Figure 1), and E83.1 (disorders of iron metabolism) for hemochromatosis only if associated with at least 3 therapeutic phlebotomies (codes are described in Figure 1) over a 12-month period.

#### Deaths Related to Main CLD, PLC, or Rare Liver Diseases

As some CLDs are only identified at the time of death, the fourth possible inclusion condition was the presence of any of the *ICD-10* codes listed above for main CLD, PLC, and rare liver diseases (excluding the nonspecific ones E83.1 and K83.0 as mentioned above) in the medical causes of death database in the SNDS (derived from CépiDc).

### Inclusion Period and Follow-Up

ESND beneficiaries were included in the RESONANCE cohort as “2015-2021 incident cases” if they presented at least one condition suggesting the presence of a CLD according to the multicriteria algorithm detailed in the previous section, with the first occurrence identified from January 1, 2015, to December 31, 2021.

If ESND beneficiaries presented at least one condition of the targeting algorithm between January 1, 2013, and December 31, 2014, they were included in the cohort as “prevalent cases over the 2015-2021 period.” The initial identification code—the one that granted the patient entry into the cohort—corresponds to the inclusion (or index) date.

For each case, information on medical history and events of interest was retrieved from January 1, 2013, up to death or until December 31, 2023 (whichever came first). The minimal follow-up period was 2 years (Figure 2).

**Figure 2:**
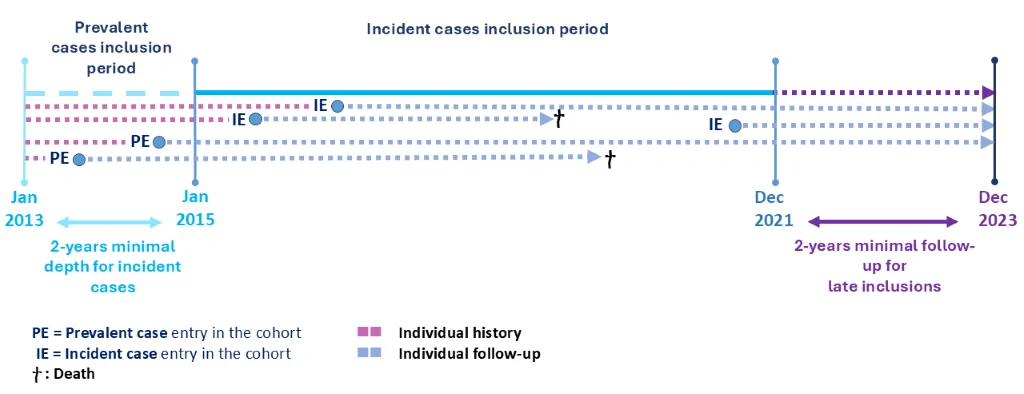
Inclusion period, follow-up period, and case definitions in the RESONANCE cohort.

### Data Collection (Variables and Definitions)

Extensive information about patient characteristics, comorbidities, and medical management has been collected from SNDS in PMSI, DCIR, and CépiDc tables as summarized in Figure 3.

**Figure 3:**
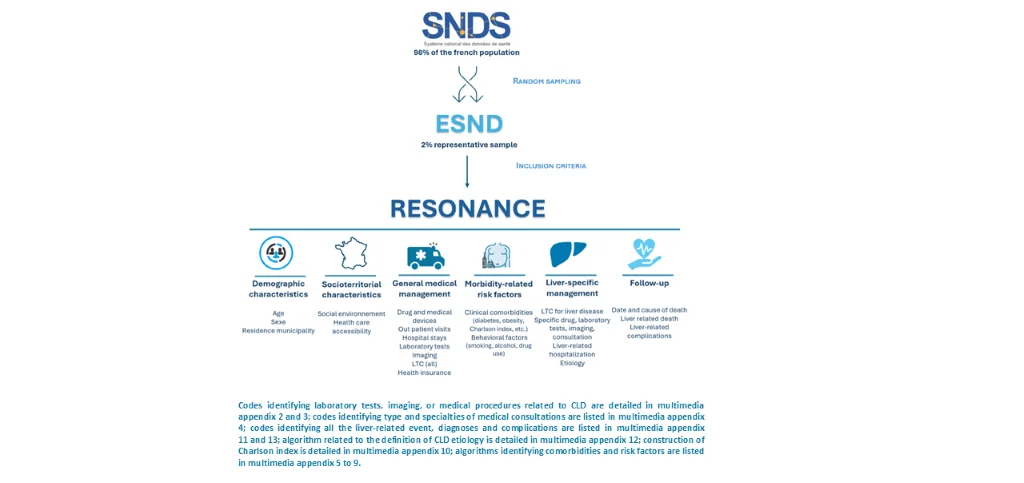
Study cohort design and collected data. CLD: chronic liver disease; ESND: a 2% representative sample from SNDS (Échantillon du Système National des Données de Santé); LTC: long-term condition (Affection Longue Durée); SNDS: Système National des Données de Santé (National Health Data System).

All codes, sources, and period of search used are detailed in Multimedia Appendices 2 to 14.

### Medical Management

For each patient, we collected the following data on medical management and follow-up: hospitalizations stays and consultations, outpatient visits to the general practitioner, nonhospital medical consultations to gastrointestinal (GI) specialists as well as specialists involved in the management of risk factors for CLD and comorbidities (cardiovascular pathology, internal medicine, pediatrics, pulmonology, physical medicine and rehabilitation, psychiatry, geriatrics, nephrology, endocrinology, and vascular medicine), liver diseases–related imaging and therapeutic procedures, biologic tests of interest (aspartate aminotransferase [AST], alanine aminotransferase [ALT], and platelets count; Multimedia Appendices 2 to 4). As the type of specialist is not available in case of outpatient visits conducted in hospital facilities, we used 2 proxies to identify GI consultations in the hospital setting: (1) all hospital consultations and (2) hospital consultations coupled with liver imaging or endoscopy or elastography or liver biopsy or transjugular intrahepatic portosystemic shunt (TIPS) within 6 months before or after.

### Identification of Comorbidities and Risk Factors

Comorbidities of interest (diabetes, obesity, sleep apnea syndrome, dyslipidemia, arterial hypertension, metabolic syndrome, and HIV infection) and unhealthy behaviors (alcohol misuse, tobacco or drug use) identification was based on proxy algorithms using hospital diagnoses, LTC, procedures, medications, and reimbursed health care data. Detailed definitions and coding algorithms are provided in the Multimedia Appendices 5 to 9.

The Charlson Comorbidity Index (CCI) at the time of inclusion (Multimedia Appendix 10), assessing the level of comorbidity according to 17 predefined comorbid conditions, was computed according to previous studies [45-50]. As all patients in the RESONANCE cohort display a liver disease, the CCI was always ≥1 to ensure that CLD was automatically recorded as liver comorbidity. The use of a modified CCI excluding liver-related conditions will also allow a more accurate assessment of extrahepatic baseline comorbidities [51].

### CLD Causes, Main Etiology, Complications, and Severity

CLD causes (viral, alcoholic, metabolic, and rare diseases) have been defined according to combinations of *ICD-10* codes, procedure codes, medication codes, or biology codes as detailed in Multimedia Appendix 11. Algorithms regarding CLD causes are consistent with previous studies conducted in the SNDS [52-59].

Main etiology of the CLD was categorized into mutually exclusive classes (Multimedia Appendix 12): ALD, viral B or C hepatitis-related liver disease (Viral), rare disease–related liver disease (RD), MASLD, metabolic dysfunction–associated alcohol-related liver disease (MetALD), other combinations of causes referred to as mixed causes of liver disease (MD) and undetermined when none of the previous etiologies were identified.

Cirrhosis and liver-related complications, including major clinical events reflecting disease progression, such as ascites, hepatic insufficiency, portal hypertension, hepato-renal syndrome, and refractory encephalopathy, were identified using *ICD-10* and procedure codes listed in Multimedia Appendix 13. PLC (identified through *ICD-10* codes) was also considered as a complication.

Liver transplantation was recorded using *ICD-10* code and procedure code (Multimedia Appendix 2).

All-cause mortality was recorded through the date of death in various tables in SNDS (PMSI, DCIR, and CépiDc). When available, liver-related mortality will also be explored based on *ICD-10* codes of main or associated causes of death recorded in the CépiDc tables implemented in the SNDS.

### Access to Health Care and Social Environment

#### Health Care Coverage

Information on beneficiaries’ health insurance coverage was recorded. Universal health coverage (named CMU “[couverture maladie universelle]” before 2016 and “[protection universelle maladie]” PUMA since 2016) was collected as a health insurance status variable. Complementary universal health coverage (CMU-C) before 2019, solidarity-based complementary health coverage (Complémentaire santé solidaire; CSS) since 2019 and state medical aid (Aide médicale de l’État [AME]) were considered individual-level markers of social vulnerability.

Allocation of 100% reimbursement of expenditure related to LTC was recorded. Eligible conditions are registered on a list of 30 groups of major chronic illnesses, including liver disease (chronic active liver disease [hepatitis B or C] and cirrhosis), metabolic comorbidities such as diabetes, and cancer (list updated in Decree Number 2011-77 of January 19, 2011 [Official Journal of January 21, 2011]) [60]. Registration of a referring practitioner was also collected.

#### Social Environment of the Municipality of Residence

A geographical social disadvantage index allocated by municipality of residence, the French Deprivation Index (FDep Index) [61], combining census data about income, unemployment, educational level, and low-skilled jobs at the municipality level is available from the CNAM mapping. It is categorized into quintiles, the highest being the most deprived.

#### Accessibility to Primary Care of the Municipality of Residence

Access to health care was assessed using the Localized Potential Accessibility (Accessibilité Potentielle Localisée [APL]), measuring the accessibility to primary care at the municipality level (estimating geographic access as well as the supply and demand relative to general practitioners). Accessibility was considered as low if APL was under 2.5, intermediate between 2.5 and 4, and good if superior to 4 [62].

### End Points

End points will be properly described in each upcoming manuscript focusing on specific research questions. To assess the primary research objective, impact of nonoptimal care pathways on the incidence of severe forms of CLD, the following definitions will be used:

Severe forms of CLD encompass:

1. hospitalizations with a diagnosis of cirrhosis or cirrhosis-related complications;
2. incident PLC not receiving curative treatment within 6 months (Multimedia Appendix 14 for the corresponding treatment codes);
3. deaths from hepatic causes.

Nonoptimal care pathways (summarized in Figure 4) were defined according to both disease severity and the timing of severe form onset:

**Figure 4:**
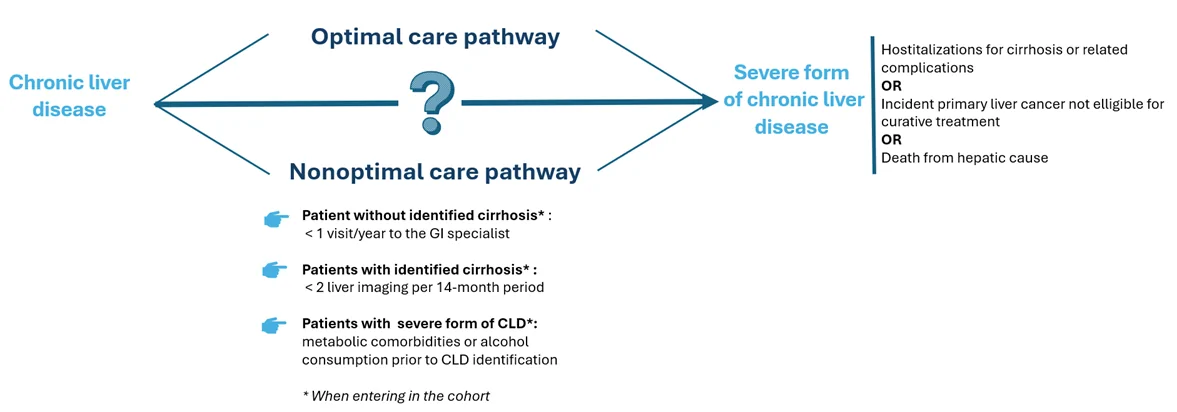
Care pathways and chronic liver disease prognosis: definitions in the RESONANCE cohort. CLD: chronic liver disease; GI: gastrointestinal.

1. in patients with CLD but without identified cirrhosis when entering the cohort: less than one visit per year to the GI specialist,
2. in patients with ambulatory cirrhosis when entering the cohort: less than 2 liver imaging (see list of codes in Multimedia Appendix 2) per 14-month period (to allow for a 1-month delay every 6-month period in the imaging schedule) [25-27], and
3. in patients entering the cohort with a severe form of CLD upfront: nonoptimal care pathway is defined by the presence of at least one metabolic-related comorbidity (obesity, diabetes, metabolic syndrome, or sleep apnea) or alcohol condition or treatment identified within 2 years prior to the onset of the inaugural severe form (index date) according to codes listed in Multimedia Appendices 5 and 7.

Secondary end points will include, but are not limited to, the following:

1. Incidence, prevalence, and mortality from CLD (2015-2021) estimated crude and age-standardized rates (per 100,000 person-years) using the ESND population as reference, stratified by type of CLD.
2. Quality of monitoring and management of CLD (2015-2023, including the COVID-19 period):
  - proportion of patients receiving optimal follow-up, and
  - temporal trends in care quality indicators before (2015-2019) and during (2020-2021) the COVID-19 pandemic, assessed by interrupted time-series analysis;
3. Sociodemographic, medical, geographic, and temporal factors associated with severe forms of CLD. Hazard ratios (from multivariable Cox proportional hazards models) for the association between the following covariates and the primary end point:
  - sociodemographic: age, sex, socioeconomic deprivation index (eg, FDep Index), health insurance status;
  - medical: CLD etiology, CCI, presence of metabolic risk factors;
  - geographic: municipality of residence; and
  - temporal: year of CLD diagnosis, calendar period (pre- and peri-COVID-19).
4. Outcomes of patients with CLD, overall and by CLD type (follow-up to December 31, 2023): 2-year overall survival and liver-specific survival as well as occurrence of liver-related events.

### Statistical Analyses

Baseline demographic, socioeconomic, clinical, and health care use characteristics will be described overall and according to CLD subtype, sex, disease severity, and other clinically relevant subgroups. Continuous variables will be summarized using means and SDs or medians and IQRs, as appropriate, whereas categorical variables will be presented as counts and percentages. Comparisons between groups will be performed using appropriate parametric or nonparametric tests according to variable type and distribution, including chi-square tests or Fisher exact tests when sample sizes are small for categorical variables and Student *t* tests or Wilcoxon rank-sum tests for continuous variables, as appropriate. The distribution of major liver disease etiologies, risk factors, comorbidities, health care use indicators, and social deprivation measures will be specifically examined.

Due to the medico-administrative nature of the data, missing data are expected to be limited and concern only social deprivation measured through the FDep Index (3.8%), health care accessibility measured through the APL index (1.7%), and causes of death (8.5%). Given the low proportion of missing data and the administrative nature of the database, complete-case analyses will usually be performed without multiple imputation. Multiple imputation may nevertheless be considered for specific research questions or sensitivity analyses if the extent or pattern of missingness justifies it. All other study variables are complete.

Incidence, prevalence, and mortality rates will be estimated overall with their corresponding 95% CI, based on large-sample normal approximation and stratified according to age, sex, social deprivation (FDep Index), health care accessibility (APL), and French departments. Whenever appropriate, rates will be age-standardized using the World Health Organization (WHO) World Standard Population.

Time-to-event analyses will be conducted to assess the association between care pathways and the occurrence of severe forms of CLD. The time origin will be the date of cohort entry, and patients will be followed until the first event of interest, death, loss to follow-up, or administrative censoring. Survival probabilities will be estimated using Kaplan-Meier methods and compared using log-rank tests for outcomes without competing risks.

Multivariable Cox proportional hazards models will be used to estimate adjusted hazard ratios. Age, sex, social deprivation, and major clinical characteristics, including liver disease etiology, risk factors, comorbidities, and disease severity indicators, will be systematically considered as potential confounders. The proportional hazards assumption will be assessed using Schoenfeld residuals through the cox.zph() function in R (R Foundation for Statistical Computing). In the presence of nonproportional effects, alternative modeling approaches such as time-dependent Cox models using the timecox() function will be considered.

For analyses involving time-varying exposures or covariates, such as treatment initiation, disease progression, incident PLC, liver transplantation, cirrhosis decompensation, or changes in health care use during follow-up, appropriate longitudinal modeling strategies incorporating time-dependent variables will be applied. Depending on the research question and data structure, additional analytical approaches adapted to longitudinal data, including time-series analyses, may also be considered.

Competing risks will be explicitly considered when studying outcomes such as liver-related death, cirrhosis decompensation, PLC, or liver transplantation. Depending on the research question, cause-specific hazard models and Fine and Gray subdistribution hazard models will be used.

Potential collinearity between explanatory variables will be assessed using variance inflation factors (VIFs) and Cramer V statistics and considered.

Sensitivity analyses will explore the robustness of findings, including alternative definitions of exposures and outcomes, restrictive and expanded disease-identification algorithms, subgroup analyses, and restricted population studies. For example, the contribution of enhanced identification algorithms is illustrated in Multimedia Appendix 15.

Statistical significance will be assessed using a 2-sided alpha level of .05. All statistical analyses will be conducted using R software (R Foundation for Statistical Computing) implemented on the secure CNAM’s SNDS online platform (version 4.4.3, with versions 4.3.3 and 4.1.2 also available).

As RESONANCE is intended to support multiple research projects addressing distinct clinical questions, detailed statistical analysis plans, including model specifications and additional methodological considerations, will be further developed and reported in dedicated methodological and results manuscripts.

## Results

### Study Status

Data extraction, cohort construction, and data management were completed in January 2025, allowing initiation of cohort analyses.

### Multisource Population Identification

In total, 26,663 incident and prevalent cases of CLD had been identified between 2015 and 2021 in the ESND, constituting the RESONANCE Cohort. CNAM mapping algorithm was the main source of inclusion in the cohort, contributing to 91.5% of case identification. The 3 other sources, beyond CNAM mapping, allowed to capture 8.5% additional cases (2262/26,663). Relative and incremental contributions of each source to CLD identification are summarized in Figure 5.

**Figure 5:**
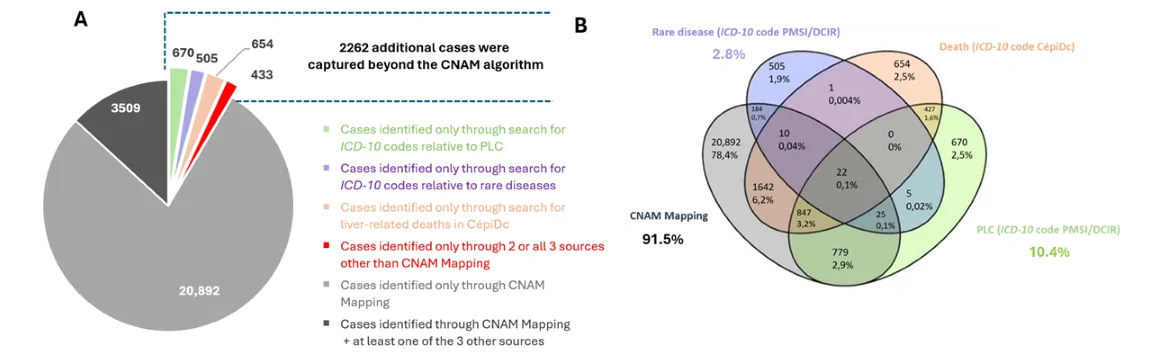
Relative and incremental contribution of each data source to chronic liver disease case identification in the cohort. (A) Contribution of each component of the targeting algorithm to the composition of the cohort. (B) Sources’ single and mutual contribution (Venn diagram). CépiDc: Centre d'épidémiologie sur les causes médicales de décès (Center for Epidemiology of Medical Causes of Death); CNAM: Caisse National d’Assurance Maladie (National Health Insurance Fund); DCIR: Données de consommations inter régime (French outpatient healthcare claims database); ICD-10: International Statistical Classification of Diseases, Tenth Revision; PLC: primary liver cancer; PMSI: Programme de médicalisation des systèmes d’information (French hospital discharge database).

### Study Population Main Characteristics

The RESONANCE cohort includes 19,645 incident and 7018 prevalent cases of CLD identified between 2015 and 2021. Preliminary descriptive analyses are shown in Table 1. The cohort encompasses a broad spectrum of etiologies, including ALD (7580/26,663, 28.4%), metabolic-associated liver disease (6030/26,663, 24.8%), viral hepatitis (3450/26,663, 12.9%), rare diseases (839/26,663, 3.1%), and mixed (2895/26,663, 10.9%) or unknown causes (5297/26,663, 19.9%). The cohort is characterized by an unbalanced sex distribution with 58.1% of men and a mean age of 59.9 (SD 17.4) years, consistent with previous reports [63,64]. During the entire follow-up (from January 1, 2015, to December 31, 2023), there were 10,216 deaths, reflecting the severity of the underlying conditions. At baseline, 43% (11,461/26,663) of the patients lived in a socially deprived environment (according to the 4th and 5th quintiles of the FDep Index), and most of the patients had good or intermediate access to primary care (23,726/26,663, 89%).

**Table 1:** RESONANCE population description.

Characteristics					Prevalent cases		Incident cases		Total
Total participants, n (%)					7018 (26.3)		19,645 (73.7)		26,663 (100)
**Demography**									
	**Sex, n (%)**								
		Male		4241 (60.4)		11,249 (57.3)		15,490 (58.1)	
		Female		2777 (39.6)		8396 (42.7)		11,173 (41.9)	
	Age at CLD^a^ diagnosis, mean (SD)			58.2 (14.8)		60.5 (18.2)		59.9 (17.4)	
	Death over follow-up, n (%)			2438 (34.7)		7778 (39.6)		10,216 (38.3)	
	Hepatic cause of death over follow-up, n (%)			1084 (44.5)		2811 (36.1)		3895 (38.1)	
	Unknown cause of death, n (%)			237 (9.7)		633 (8.1)		870 (8.5)	
	Length of follow-up, mean (SD)			105.5 (35.5)		45.6 (33.2)		61.4 (42.9)	
	Length of observation before CLD diagnosis, mean (SD)			24 (0)		64.6 (24.2)		53.9 (27.4)	
**Causes identified over follow-up, n (%)**									
	MASLD^b^ (large version)			733 (10.4)		6030 (30.7)		6763 (25.4)	
	ALD^c^ or MetALD^d^ (large version)			2915 (41.5)		6071 (30.9)		8986 (33.7)	
	**Viral hepatitis identified**			1603 (22.8)		1191 (6.1)		2794 (10.5)	
		Viral hepatitis C		2497 (35.6)		1552 (7.9)		4049 (15.2)	
		Viral hepatitis B (all)		853 (12.2)		995 (5.1)		1848 (6.9)	
		Viral hepatitis B (with delta)		63 (0.9)		52 (0.3)		115 (0.4)	
	Rare disease (all)			587 (8.4)		903 (4.6)		1490 (5.6)	
	Hemochromatosis			169 (2.4)		367 (1.9)		536 (2)	
	Wilson disease			26 (0.4)		17 (0.1)		43 (0.2)	
	Primary biliary cholangitis			238 (3.4)		156 (0.8)		394 (1.5)	
	Autoimmune hepatitis			121 (1.7)		168 (0.9)		289 (1.1)	
	Primary sclerosing cholangitis			49 (0.7)		119 (0.6)		168 (0.6)	
	Budd-Chiari syndrome			19 (0.3)		40 (0.2)		59 (0.2)	
	Other			13 (0.3)		84 (0.4)		107 (0.4)	
**Main etiology identified over follow-up** **, n (%)**									
	ALD			978 (13.9)		2671 (13.6)		3649 (13.7)	
	MASLD			572 (8.2)		6030 (30.7)		6602 (24.8)	
	MetALD			1105 (15.7)		2826 (14.4)		3931 (14.7)	
	Mixed cause			1592 (22.7)		1303 (6.6)		2895 (10.9)	
	Rare disease alone			304 (4.3)		535 (2.7)		839 (3.1)	
	Viral cause alone			1861 (26.5)		1589 (8.1)		3450 (12.9)	
	Unknown			606 (8.6)		4691 (23.9)		5297 (19.9)	
**Risk factors identified over follow-up, n (%)**									
	Alcohol-related condition or treatment			2766 (39.4)		6071 (30.9)		8837 (33.1)	
	Tobacco-related condition or treatment			2841 (40.5)		7509 (38.2)		10,350 (38.8)	
	Obesity			1337 (19.1)		5140 (26.2)		6477 (24.3)	
	Type 2 diabetes			1437 (20.5)		4892 (24.9)		6329 (23.7)	
	Hypertension			3954 (56.3)		11,485 (58.5)		15,439 (57.9)	
	Dyslipidemia			1630 (23.2)		6031 (30.7)		7661 (28.7)	
	Metabolic syndrome			724 (10.3)		2762 (14.1)		3486 (13.1)	
	Sleep apnea syndrome			612 (8.7)		2449 (12.5)		3061 (11.5)	
	Primary liver cancer			932 (13.3)		2032 (10.3)		2964 (11.1)	
**Charlson score when entering the cohort, mean (SD)**									
	Standard (with liver diseases)			3 (2.7)		4.3 (3.3)		4 (3.2)	
	Modified (excepting liver diseases)			1.3 (2.1)		2.3 (2.7)		2 (2.6)	
**Social environment and medical management at inclusion, n (%)**									
	**French Deprivation Index quintile of the municipality of residence**								
			1 (least deprived)	1259 (17.9)		3217 (16.4)		4476 (16.8)	
			2	1210 (17.2)		3410 (17.4)		4620 (17.3)	
			3	1441 (20.5)		3651 (18.6)		5092 (19.1)	
			4	1372 (19.5)		4019 (20.5)		5391 (20.2)	
			5 (most deprived)	1533 (21.8)		4537 (23.1)		6070 (22.8)	
			—^e^	203 (2.9)		811 (4.1)		1014 (3.8)	
	**Access to primary care of the municipality of residence**								
			Low	519 (7.4)		1967 (10)		2486 (9.3)	
			Intermediate	2635 (37.5)		8171 (41.6)		10,806 (40.5)	
			Good	3728 (53.1)		9192 (46.8)		12,920 (48.5)	
			— ^e^	136 (1.9)		315 (1.6)		451 (1.7)	
	LTC^f^ full coverage except CLD			3393 (48.3)		11,658 (59.3)		15,051 (56.4)	
	Referring physician declared			4033 (57.5)		14,593 (74.3)		18,626 (69.9)	
**Medical management during follow-up, n (%)**									
	LTC full coverage for CLD			1880 (26.8)		3250 (16.5)		5130 (19.2)	
	Referring physician declared (at least one)			6437 (91.7)		17,637 (89.8)		24,074 (90.3)	

^a^CLD: chronic liver disease.

^b^MASLD: metabolic dysfunction–associated steatotic liver disease.

^c^ALD: alcohol-related liver disease.

^d^MetALD: metabolic dysfunction–associated alcohol-related liver disease.

^e^ Not available.

^f^LTC: long-term condition.

### Impact of Definitions on Algorithm Performance

As detailed in Multimedia Appendix 15, the choice of algorithm definitions substantially influenced the estimated prevalence of each CLD subtype. Cirrhosis was identified in 42.5% (11,332/26,663) of patients when relying solely on *ICD-10* cirrhosis codes, rising to 50.6% (13,488/26,663) of patients when an expanded algorithm incorporating cirrhosis-related complications was applied—representing a relative increase of 19%. Similarly, MASLD identification increased from 5500 to 6763 cases when the definition was broadened beyond *ICD-10* codes K75.8 and K76.0 to additionally require the presence of metabolic risk factors in the absence of competing etiologies, yielding 1263 additional cases (+23%). For ALD, extending the definition beyond *ICD-10* code K70 to encompass alcohol-related comorbidities and alcohol cessation treatments increased the total number of patients from 6146 to 8986 (+46.2%). Taken together, these findings underscore the sensitivity of epidemiological estimates to the practical definitions applied and highlight the importance of algorithm selection when conducting database studies on CLD, beyond the French setting.

## Discussion

RESONANCE is an original cohort including 26,663 incident and prevalent cases of CLD identified between 2015 and 2021 in the 2% representative sample of the SNDS. The main targeting algorithm accounted for 91.5% of case identification, while complementary data sources provided 8.5% additional cases. Preliminary analyses also demonstrated substantial variability in epidemiological estimates according to the operational definitions and algorithms used for CLD identification.

The RESONANCE cohort is expected to generate comprehensive, nationwide real-world evidence on CLD, providing detailed estimates of incidence, prevalence, and mortality across different types of CLD and disease stages. Its longitudinal design will enable the reconstruction of health care trajectories over time, including prediagnosis phases, patterns of care use, and transitions between health care settings. The cohort will allow identification of determinants of delayed diagnosis, disease progression, and access to curative treatments, particularly in PLC. In addition, the integration of socioeconomic, geographic, and health care access indicators will support the analysis of gender, social, and territorial disparities in disease presentation and outcomes. These findings are expected to inform both clinical practice and public health policies by identifying vulnerable populations and guiding targeted interventions. A primary strength of the RESONANCE study lies in its use of the SNDS. As one of the largest health administrative databases in the world, it provides near-exhaustive population coverage, capturing approximately 98%-99% of the French population. Data within the SNDS are standardized, quality-controlled, and validated through a rigorous transmission standards protocol, ensuring a high level of reliability and completeness. For the purposes of the RESONANCE project, analyses were conducted using the 2% representative sample ESND to ensure feasibility and methodological robustness while avoiding the substantial technical, computational, and environmental burden associated with full database extraction given the large number of CLD cases, the extensive set of variables collected, and the breadth of the study period spanning nearly a decade of historical and prospective follow-up (2015-2023). This approach is aligned with French regulatory recommendations promoting data minimization and with CNAM guidance encouraging the use of the ESND whenever appropriate. Moreover, previous studies based on the previous sample (Échantillon Généraliste des Bénéficiaires [EGB]—a representative 1/97th random sample of the SNDS) have demonstrated the validity of this approach [40,65,66].

A further strength of the SNDS is its comprehensive capture of health care expenditure and use across all care settings, encompassing both inpatient and outpatient data. This represents a substantial methodological advantage over studies restricted to hospital-based databases such as the PMSI. Building on this, a key distinguishing feature of RESONANCE lies in its longitudinal nationwide design and its ability to reconstruct complete patients’ health care trajectories over time, including the prediagnostic period. Several national cohorts, including the French viral- or ALD-related cirrhosis cohorts CirVir and CIRRAL [12,67], as well as French PLC cohorts such as CHANGH, CHIEF, CRB, and ACABi [22,68-70], international cohorts including the Veterans Affairs cohorts [71], the Texas HCC Consortium [72], and TARGET-HCC [73], have provided key insights into CLDs. However, such cohorts are generally restricted to selected populations in high-volume centers or specific disease stages, limiting their generalizability to the whole CLD population encountered in routine clinical practice. Information on risk factors or medical management prior to diagnosis is usually limited. In contrast, RESONANCE adopts a nationwide, representative population-based approach that overcomes these limitations in real-world conditions. Its design enables the characterization of care pathways across public and private sectors, and across high- and low-volume centers, and over extended follow-up periods, offering a uniquely dynamic and granular view of disease progression and management.

Beyond the epidemiological contributions, the RESONANCE study carries substantial methodological value. Its case identification is built upon the CNAM identification algorithm, which currently represents the national benchmark in France for estimating health care costs and monitoring medical activity related to liver diseases—albeit with the notable limitation of excluding PLC and rare liver conditions. In RESONANCE, this fundamental framework is substantially extended through a multisource identification algorithm that combines complementary SNDS data sources, including hospital discharge data, LTC status, outpatient care claims, procedures, drug dispensing data, and cause-of-death information. This multisource strategy offers a meaningful improvement in case ascertainment and enables the identification of patients across all stages of CLD—including those managed exclusively in ambulatory settings or captured solely through mortality data—who would otherwise remain undetected. The algorithm developed within RESONANCE may therefore serve as a methodological reference for future administrative data–based studies on CLD in France and beyond. It provides a transparent foundation for future studies derived from this cohort and is expected to support numerous subsequent epidemiological, ecological, prognostic, and health services research analyses.

In addition, RESONANCE incorporates data on patients’ social environment and health care accessibility at the municipality level—a level of contextual detail that has not been available in historical CLD cohorts. By integrating these sociogeographic variables alongside individual-level clinical data, RESONANCE is positioned to generate critical and methodologically innovative insights into the determinants of health trajectories in CLD. These findings are expected to inform corrective actions in routine clinical practice and guide the development of targeted public health interventions for vulnerable populations, in line with the principle of proportionate universalism [74], with the ultimate aim of reducing care disparities and improving equitable access to care across France.

Taken together, this comprehensive scope positions RESONANCE as a uniquely suited framework for generating nationally representative epidemiological estimates and informing evidence-based public health strategies for CLD in France.

However, research leveraging the SNDS has limitations. The first stems from the data source itself. SNDS lacks physical examination data, laboratory or imaging results, sometimes limiting the precise assessment of clinical stages of CLD and does not contain individual social status (only defined at the municipality level). Assessment of treatment duration can be challenging as treatment compliance is not captured. Prescribed dosage, consumption of over-the-counter medication, or drugs received through a clinical trial and even chemotherapy treatments are lacking, and in-hospital treatments can be identified only if qualified in the “en sus” list (beyond the hospitalization fees) [30].

Moreover, case identification algorithms rely on coding of liver diseases, risk factors, and comorbidities. However, exhaustivity of coding is highly dependent on resources dedicated to coding in health care facilities, exposing to a risk of under ascertainment due to incomplete or inconsistent coding practices. This limitation is particularly relevant for comorbidities and behavioral risk factors, which are systematically undercaptured in administrative data. Conditions such as obesity, sleep apnea, alcohol misuse, tobacco use, or drug use are likely to be underdetected, and corresponding variables should therefore be interpreted as proxy indicators of clinical exposure rather than exhaustive population-level measures. Alcohol misuse is a salient example: its identification in administrative data relies on indirect markers, including alcohol-related diseases or psychiatric conditions and prescriptions for withdrawal treatment, which inevitably fail to capture a substantial proportion of at-risk individuals with more moderate consumption. A related structural limitation concerns the availability of *ICD-10* diagnostic codes across care settings. In the SNDS, *ICD-10* codes are systematically recorded in hospital discharge summaries, but their availability in outpatient settings is restricted to 2 specific contexts: when an LTC status has been formally granted, or when specific treatments, biology, or procedures—primarily applicable to chronic viral hepatitis B and C—are performed outside of hospital. Therefore, nonhospitalized patients with nonviral, noncirrhotic liver diseases are at risk of remaining unidentified within the SNDS, which may introduce a systematic bias toward the overrepresentation of more severe or advanced disease forms in prevalence estimates, and should be considered when interpreting epidemiological findings from this study. Prevalence estimates derived from SNDS data should be interpreted as a minimum, health care–based or “identified” prevalence, reflecting diagnosed and recorded disease within the health care system rather than true underlying population prevalence.

The second limitation concerns the restricted availability of fully validated case identification algorithms for CLD in administrative data. This challenge is common to administrative database studies both nationally and internationally, where algorithms may vary considerably across settings and research groups, particularly in the field of hepatology [44,75,76]. This variability reflects the lack of universally accepted definitions for many liver conditions, including cirrhosis and its etiologies, which remains an ongoing challenge in the field [77-79]. In line with standard practices in large-scale real-world studies, our algorithms for identifying CLD subtypes, risk factors, and comorbidities were based on published definitions whenever available [2,44,47-50,52-59,80]. For CLD, the foundational framework was derived from the CNAM liver disease mapping algorithm, which to date has been formally validated only for viral hepatitis B and C cohorts [52]. If published algorithms were unavailable at the time of study conception, previously unpublished—or subsequently published, as for PSC algorithms [44] were used or algorithms were specifically developed for the purposes of this study, as was the case for hereditary hemochromatosis. Importantly, in that case, all algorithms were derived through a structured, literature-based, and expert-driven consensus process involving both hepatology specialists and medico-administrative data experts, ensuring that case definitions were clinically grounded and methodologically robust despite the inherent constraints of administrative data. All the algorithms of the RESONANCE study have been explicitly detailed in the Multimedia Appendices 2 to 15 to ensure reproducibility and facilitate methodological comparisons with future studies. To address these limitations, sensitivity analyses will be conducted using alternative algorithm definitions as well as analyses restricted to subgroups identified through more stringent and robust criteria, in order to assess the robustness of findings. Furthermore, a dedicated validation study is planned as a separate research project, leveraging hospital clinical data warehouses to enable direct verification of diagnoses against individual patient records. This initiative will provide formal estimates of the sensitivity, specificity, and positive predictive value of the algorithms developed within RESONANCE, thereby strengthening the methodological foundation of both this study and future administrative data–based research on CLD in France.

Importantly, the primary objective of this study is to investigate associations and disease trajectories. In this context, nondifferential misclassification—while possible—is expected to bias estimates toward the null rather than generate spurious associations. In addition, outcome misclassification is likely to be more limited for severe events such as decompensated cirrhosis, PLC, or death, which are more consistently captured in the SNDS.

A final limitation concerns the completeness of cause-of-death data, since they were unavailable for 8.5% (870/10,216) of deceased patients, primarily attributable to incomplete record linkage between SNDS and the CépiDc source database. Additional missingness may arise from deaths occurring outside France (not captured within the SNDS), but this situation is likely to be negligible. Furthermore, throughout the study period, paper-based certification remained the predominant modality, although the proportion of electronic certificates increased progressively from approximately 10%-15% in 2015 to 30%-32% in 2021 [81,82]. This gradual transition toward electronic certification may have introduced a degree of temporal heterogeneity in the completeness and accuracy of cause-of-death ascertainment, which should be considered when interpreting trends in liver-related mortality over the study period.

Despite these limitations, the RESONANCE cohort provides a unique and valuable resource for generating comprehensive, nationwide, representative real-world evidence on CLD encompassing their etiology, health care trajectories, and multifactorial determinants. RESONANCE is positioned to deliver new scientific knowledge and actionable insights that can meaningfully inform both clinical practice and evidence-based public health policies for chronic liver disease in France.

## Appendix

Multimedia Appendix 1 High resolution version of Figure 1.

Multimedia Appendix 2 Liver related imaging and procedures available in RESONANCE (CCAM codes).

Multimedia Appendix 3 Biology tests of interest available in RESONANCE (NABM codes).

Multimedia Appendix 4 Type (a) and Specialties (b) of medical consultations.

Multimedia Appendix 5 Identification algorithm for metabolic comorbidities and sleep apnea.

Multimedia Appendix 6 HIV identification algorithm.

Multimedia Appendix 7 Alcohol consumption identification algorithm.

Multimedia Appendix 8 Tobacco consumption identification algorithm.

Multimedia Appendix 9 Drug use identification algorithm.

Multimedia Appendix 10 Algorithm for Charlson comorbidities index.

Multimedia Appendix 11 Identification algorithms for all possible Chronic Liver Disease (CLD) causes.

Multimedia Appendix 12 Identification algorithms for main etiology of Chronic Liver Diseases (CLD).

Multimedia Appendix 13 Identification algorithm for severity of CLD and complications.

Multimedia Appendix 14 Liver cancer treatments.

Multimedia Appendix 15 Contribution of enhanced algorithms to CLD identification.

## Abbreviations

AFP: alpha-fetoprotein
AIH: autoimmune hepatitis
ALD: alcohol-related liver disease
ALT: alanine aminotransferase
AME: Aide médicale de l’État (state medical aid)
APL: Accessibilité Potentielle Localisée (Localized Potential Accessibility)
AST: aspartate aminotransferase
CCI: Charlson Comorbidity Index
CépiDc: Centre d'épidémiologie sur les causes médicales de décès (Center for Epidemiology of Medical Causes of Death)
CLD: chronic liver disease
CMU: universal health coverage
CMU-C: complementary universal health coverage
CNAM: Caisse National d’Assurance Maladie (National Health Insurance Fund)
CNIL: Commission Nationale de l’Informatique et des Libertés (French Data Protection Authority)
CSS: Complémentaire santé solidaire (complementary health coverage)
DCIR: Données de consommations inter régime (French outpatient healthcare claims database)
EASL: European Association for the Study of the Liver
EGB: Échantillon Généraliste des Bénéficiaires
ESND: Échantillon du Système National des Données de Santé (Simplified Sample of the French National Health Data System)
FDep Index: French Deprivation Index
Francim: French Network of Cancer Registries
GI: gastrointestinal
HBV: hepatitis B virus
HCC: hepatocellular carcinoma
HCV: hepatitis C virus
ICD-10: International Statistical Classification of Diseases, Tenth Revision
IRB: institutional review board
LTC: long-term condition (Affection Longue Durée)
MASLD: metabolic dysfunction–associated steatotic liver disease
MetALD: MASLD associated with alcohol-associated liver disease
MD: mixed causes of liver disease
PBC: primary biliary cholangitis
PLC: primary liver cancer
PMSI: Programme de médicalisation des systèmes d’information (French hospital discharge database)
PSC: primary sclerosing cholangitis
RD: rare disease–related liver disease
SNDS: Système National des Données de Santé (National Health Data System)
SNIIRAM: Système National d'Information Inter Régimes de l'Assurance Maladie
TIPS: transjugular intrahepatic portosystemic shunt
UDCA: ursodeoxycholic acid
VIF: variance inflation factor
Viral: viral B or C hepatitis-related liver disease
WHO: World Health Organization

## Data Availability

The datasets generated or analyzed during this study are not publicly available due to French legal restrictions governing access to the French National Health Data System (SNDS) but may be accessed by eligible researchers subject to authorization from the relevant French authorities.
