# Supplementary material for: RESONANCE: Protocol for a Nationwide Cohort Study of Chronic Liver Diseases Using the French National Health Data System
Source: JMIR Res Protoc. 2026 Oct 5;15:e89577. doi: 10.2196/89577 (PMC13680907; doi:10.2196/89577)
Supplement: Supplementary file 1 [file resprot_v15i1e89577_app1.pdf]

SNDS

ESND 2% representative sample

RESONANCE COHORT

at least one of the 4 following conditions:

CNAM Mapping 11th category « Liver diseases »	*ICD-10* codes (PMSI or LTC)	*ICD-10* codes (PMSI or LTC)	*ICD-10* codes (CépiDc)
	**Primary liver cancer = C22**	**Rare and chronic liver diseases not included in the CNAM mapping**	Deaths from: **Liver diseases**, **Primary liver cancer**, **Rare diseases**

Patients identified by the "Liver or pancreas disease" identification algorithm from the **CNAM mapping** of pathologies and expenditure **(SUP_HFOI_IND)** for the years 2013 to 2021 **AND** for whom, between 2013(1) and 2021, one of the following criteria can be found in all the **SNDS tables**:

▶ a **hospitalization** entered in one of the **PMSI** tables (via any type of diagnosis) with one of the following ***ICD-10*** codes (digits in 2 or 3 positions):

- B18 (chronic viral hepatitis)
- I85 (esophageal varices)
- K70 (alcoholic related liver disease)
- K71 (toxic liver disease)
- K72 (liver failure not elsewhere classified)
- K73 (chronic hepatitis not elsewhere classified)
- K74 (fibrosis and cirrhosis of the liver, including rare disease code K743 Primary biliary cholangitis (PBC))
- K75 (other inflammatory liver diseases, including rare disease code K754 Autoimmune hepatitis (AIH))
- K76 (other liver diseases, including rare disease codes K764 Peliosis hepatis and K765 Veno-occlusive disease of the liver) except K76.1 (of cardiac origin))
- Z94.4 (liver transplant)

▶ **a long-term condition (LTC)** with one of these same ***ICD-10*** codes (identification possible before 2013)

▶ delivery of **treatment for viral hepatitis** (identified by ATC, CIP and UCP codes)

- adefovir, dipivoxil, telbivudine, zeffix on 3 different dates,
- interferon and ribavirin at the same date,
- or direct-acting HCV antiviral: telaprevir, boceprevir, simeprevir, daclatasvir, ledipasvir, velpatasvir, sofosbuvir, glecaprevir, pibrentasvir, dasabuvir, ombitasvir, paritaprevir, ritonavir, ekbasvir, grazoprevir

▶ at least **1 HCV genotyping** reimbursement (NABM code 4125),

▶ at least 3 reimbursements for **HCV RNA PCR** (NABM code 4124) **OR** at least 2 reimbursements for **HCV RNA PCR** combined with at least one **hepatic fibrosis** evaluation (NABM codes 1000 1001 1002 or CCAM codes HLQM002, HLHB001, HLHH001, HLHH005, HLHJ003).

Patients identified with a **C22 *ICD-10*** code (in any **PMSI** table or **DCIR** table via LTC) for **primary liver cancer** between 2013(1) and 2021

- C22 Primary liver cancer
- C22.0 Hepatocellular carcinoma
- C22.1 Malignant tumor of the intra-hepatic bile ducts
- C22.2 Hepatoblastoma
- C22.3 Angiosarcoma
- C22.4 Other sarcoma
- C22.7 Other carcinoma
- C22.8 Unspecified primary malignant liver tumor
- C22.9 Unspecified liver cancer primary ou secondary

Patients identified by an ***ICD-10*** code (in any **PMSI** table or **DCIR** table via LTC) for a **rare chronic liver disease not included in the CNAM mapping** between 2013(1) and 2021

- E831 Hemochromatosis(2)
- E830 Wilson's disease
- K830 Primary sclerosing cholangitis (CSP)(3)
- I820 Budd-Chiari Syndrom
- Q442 Bile ducts atresia

Patients identified by a **death (CépiDc** tables) between 2015 and 2021, for whom one of the cause (initial or associated) was related to an ***ICD-10*** code **of**:

Liver disease (see list in point 1)
Primary liver cancer C22 (see list in point 2)
Rare diseases (see list in point 3, excluding E831 and K830)

1. Research starting from 2013 will allow for the distinction between incident cases and prevalent cases from 2015 onwards.
2. with refinement by the criterion of at least 3 therapeutic bleedings (CCAM code FEJF003) over a year
3. with refinement by the search (in all PMSI and DCIR tables) for a chronic inflammatory bowel disease (*ICD-10* codes K50 K51 M054 M075, or 1 hepatobiliary imaging per year for 3 consecutive years CCAM codes ZCQN002, ZCQJ004, ZCQJ005 or ZCQN001) as well as 3 dispensations of ursodeoxycholic acid (ATC A05AA02) throughout the follow-up

ATC: Anatomical, Therapeutic and Chemical Classification, CCAM: codes for medical procedures [*Classification Commune des Actes Médicaux*], CépiDc: Center for Epidemiology of Medical Causes of Death [*Centre d'épidémiologie sur les causes médicales de décès*] CIP: The French national drug identifier for pharmaceutical presentations [*Code Identifiant de Présentation*] CLD: chronic liver disease, CSP: Primary sclerosing cholangitis, ESND: a 2% representative sample from SNDS [*Échantillon du Système National des Données de Santé*], HBV: Hepatitis B virus, HCV: Hepatitis C virus, *ICD-10: International Classification of Diseases 10th revision*, DCIR: French outpatient healthcare claims database [*Données de consommation inter régime*], LTC: long term condition [*Affection de Longue Durée*], NABM: codes for biological procedures [*Nomenclature des actes de biologie médicale*], PBC: Primary biliary cholangitis, PCR: Polymerase chain reaction PMSI: French hospital discharge database [*Programme de médicalisation des systèmes d'information*] SNDS: National Health Data System [*Système National des Données de Santé*].
