# Supplementary material for: RESONANCE: Protocol for a Nationwide Cohort Study of Chronic Liver Diseases Using the French National Health Data System
Source: JMIR Res Protoc. 2026 Oct 5;15:e89577. doi: 10.2196/89577 (PMC13680907; doi:10.2196/89577)
Supplement: Supplementary file 2 [file resprot_v15i1e89577_app10.docx]

| Medical Condition (Charlson comorbidities index components)^1^ | Considered present if at least one of the ICD-10^2^ codes listed | Weight for Charlson comorbidities index calculation^3^ |
| --- | --- | --- |
| Myocardial infarction | I21, I22, I252 | 1 |
| Congestive heart failure | I099, I110, I130, I132, I255, I420, I425, I426, I427, I428, I429, I43, I50, P290 | 1 |
| Peripheral vascular disease | I70, I71, I731, I738, I739, I771, I790, I792, K551, K558, K559, Z958, Z959 | 1 |
| Cerebrovascular disease | G45, G46, H340, I60, I61, I62, I63, I64, I65, I66, I67, I68, I69 | 1 |
| Dementia | F00-F03, F051, G30, G311 | 1 |
| Chronic pulmonary disease | I278, I279, J40-J47, J60-J67, J684, J701, J703 | 1 |
| Rheumatologic disease | M05, M06, M315, M32, M33, M34, M351, M353, M360 | 1 |
| Peptic ulcer disease | K25-K28 | 1 |
| Mild liver disease | B18, K700-K703, K709, K71 (except K71.1), K73, K74, K75 (except K75.8), K76 (except K76.1), Z944, E830, E831, I820, K830, Q442  + NABM codes 4124 4125 1000 1001 1002,  + CCAM codes HLQM002, HLHB001,HLHH001, HLHH005, HLHJ003,  + Drug codes for treatment of viral hepatitis (adefovir, dipivoxil, telbivudine,zeffix, interferon, ribarivine, telaprevir, boceprevir, simeprevir, daclatasvir, ledipasvir, velpatasvir, sofosbuvir, glecaprevir, pibrentasvir, dasabuvir, ombitasvir, paritaprevir, ritonavir, ekbasvir, grazoprevir) | 1 (set to 0 if the Charlson comorbidity index is used as a variable to adjust for non-liver-related comorbidities) |
| Diabetes without complications | E100, E101, E106, E108, E109, E110, E111, E116, E118, E119, E120, E121, E126, E128, E129, E130, E131, E136, E138, E139, E140, E141, E146, E148, E149 | 1 (set to 0 if diabetes with complication is identified) |
| Diabetes with complications | E102-E105, E107, E112-E115, E117, E122-E125, E127, E132-E135, E137, E142-E145, E147 | 2 |
| Hemiplegia or paraplegia | G041, G114, G801, G802, G81, G82, G830-G834, G839 | 2 |
| Renal disease | I120, I131, N032-N037, N052-N057, N18, N19, N250, Z490-Z492, Z940, Z992 | 2 |
| Any malignancy  (excluding metastases) | C00-C97 excluding C77-C80 | 2 (set to 0 if metastatic solid tumor is identified) |
| Moderate or severe liver disease | K704, K711, K72, K758, K765-K767, I85, I864, I982, I983, R17, R18, C22 | 3 (set to 0 if the Charlson comorbidity index is used as a variable to adjust for non-liver-related comorbidities) |
| Metastatic solid tumor | C77-C80 | 6 |
| AIDS/HIV | B20-B22, B24 | 6 |

^1^ Adapted from: ***1)*** Léandre C, Com-Ruelle L. Repérer les facteurs de risque des patients hospitalisés pour un premier épisode d’Accident vasculaire cérébral (AVC) et analyser les déterminants de sa gravité : l’apport des bases médico-administratives. Les rapports de l'IRDES n°570. 2019 ***2)*** Quan H, Sundararajan V, Halfon P, Fong A, Burnand B, Luthi JC, et al. Coding algorithms for defining comorbidities in ICD-9-CM and ICD-10 administrative data. Med Care. 2005;43(11):1130-9 ***3)*** Sundararajan V, Quan H, Halfon P, Fushimi K, Luthi JC, Burnand B, et al. Cross-national comparative performance of three versions of the ICD-10 Charlson index. Med Care. 2007;45(12):1210-5. ***4)*** Bannay A, Chaignot C, Blotière PO, Basson M, Weill A, Ricordeau P, et al. The Best Use of the Charlson Comorbidity Index With Electronic Health Care Database to Predict Mortality. Med Care. 2016;54(2):188-94. ***5)*** Elhence H, Dodge JL, Farias AJ, Lee BP. Quantifying days at home in patients with cirrhosis: A national cohort study. Hepatology. 2023 Aug 1;78(2):518-529. doi: 10.1097/HEP.0000000000000370. Epub 2023 Mar 31. PMID: 36994701; PMCID: PMC10363198.

^2^ Search for ICD-10 codes in all PMSI, DCIR and CépiDc tables, 2 years prior CLD identification and 6 months after

^3^ Calculation of Charlson index: sum of each medical condition x corresponding weight

AIDS: Acquired Immune Deficiency Syndrome, CCAM: Common Classification of Medical Procedures *[Classification Commune des Actes Médicaux]*, CépiDc: Center for Epidemiology of Medical Causes of Death *[Centre d'épidémiologie sur les causes médicales de décès],* DCIR: French outpatient healthcare claims database *[Données de consommations inter régime],* HIV: Human Immunodeficiency Virus, ICD-10: International Classification of Diseases, 10th revision, NABM: Nomenclature of medical biology procedures *[Nomenclature des actes de biologie médicale]*, PMSI: French hospital discharge database *[Programme de médicalisation des systèmes d’information]*
