# Supplementary material for: RESONANCE: Protocol for a Nationwide Cohort Study of Chronic Liver Diseases Using the French National Health Data System
Source: JMIR Res Protoc. 2026 Oct 5;15:e89577. doi: 10.2196/89577 (PMC13680907; doi:10.2196/89577)
Supplement: Supplementary file 3 [file resprot_v15i1e89577_app11.docx]

| Causes of CLD | Identification Criteria | Codes^1^ (ICD-10 / ATC / UCD / CIP / NABM / CCAM) |
| --- | --- | --- |
| HCV | ICD-10 B182 or HCV-specific treatments (interferon + ribavirin on the same day or direct-acting antiviral) or HCV-specific genotyping or ≥3 PCRs (or 2 PCRs + fibrosis evaluation) in a year | ICD-10: B182  NABM: 4124, 4125, 1000–1002  CCAM: HLQM002, HLHB001, HLHH001, HLHH005, HLHJ003  ATC: Interféron: L03AB09–11; Ribavirin: J05AB04, J05AP01;  Direct-acting antivirals: Telaprevir: J05AE11, J05AP02; Boceprevir: J05AE12, J05AP03; Simeprevir: J05AE14, J05AP05; Sofosbuvir: J05AX15, J05AP08;  Dasabuvir: J05AX16, J05AP09; Sofosbuvir + Ledipasvir: J05AP51, J05AX65; Sofosbuvir + Velpatasvir: J05AP55; Sofosbuvir + Velpatasvir + Voxilaprevir: J05AP56; Glecaprevir and Pibrentasvir: J05AX; Daclatasvir: J05AP07, J05AX14; Ombitasvir, Paritaprevir and Ritonavir: J05AX67, J05AP53; Elbasvir et grazoprevir: J05AX68, J05AP54.  UCD: Boceprevir: 9362992  CIP: Glecaprevir and Pibrentasvir: 3400930108765, 3400894287391 |
| HBV without delta | ICD-10 B181 or ≥3 HBV-specific treatments in a year | ICD-10: B181  ATC: Adefovir, dipivoxil: J05AF08; Entecavir: J05AF10, Telbivudine: J05AF11;  UCD: Zeffix®: 9212525, 9212531  CIP13: Zeffix®: 3400935196712, 3400935196941, 3400949501038 |
| HBV with delta | ICD-10 B180 or HBV-delta specific treatment | ICD-10: B180  CIP13: Bulevirtide: 3400958901881, 3400930211281, 3400894522706, 3400890005258 |
| HBV (broad) | HBV with or without delta as described above, or tenofovir treatment in the absence of HIV (see Multimedia Appendix 6 for the identification algorithm) | ATC: Tenofovir: J05AF07 |
| Unspecified chronic hepatitis | ICD-10 B188 or B189 | ICD-10: B188, B189 |
| Alcohol-related CLD | Strict definition: ICD-10 K70  Broad definition: presence of alcohol-related codes | ICD-10: K70 (strict)  Alcohol Consumption Identification Criteria (see Multimedia Appendix 7) (broad) |
| MASLD/MASH | ICD-10 K760 (MAFLD) or K758 (MASH) | ICD-10: K760, K758 |
| MASLD/MASH (large) = Metabolic CLD | MASLD/MASH (definition above) or presence of at least one metabolic risk factor (diabetes, obesity, metabolic syndrome or sleep apnea; see Multimedia Appendix 5 for definition) but no alcohol, viral hepatitis or rare disease | ICD-10: K760, K758, metabolic risk factors codes (see Multimedia Appendix 5)  Exclusion codes: HCV (see above), alcohol consumption (see Multimedia Appendix 7), or rare disease (see below) |
| Hemochromatosis | ICD-10 E831 + ≥3 therapeutic bloodletting/year | ICD-10: E831  CCAM: FEJF003 |
| Wilson's Disease | ICD-10 E830 | ICD-10: E830 |
| Primary biliary cirrhosis | ICD-10 K743 | ICD-10: K743 |
| Autoimmune hepatitis | ICD-10 K754 | ICD-10: K754 |
| Primary sclerosing cholangitis | ICD-10 K830 + chronic inflammatory bowel disease ICD-10 codes or ≥3 bowel imaging/year + ≥3 ursodeoxycholic acid over follow-up | ICD-10: K830, K50, K51, M075  CCAM: ZCQN002, ZCQJ004, ZCQJ005, ZCQN001  ATC: Ursodeoxycholic Acid: A05AA02 |
| Budd-Chiari syndrome | ICD-10 I820 | ICD-10: I820 |
| Biliary atresia | ICD-10 Q442 | ICD-10: Q442 |
| Peliosis hepatis | ICD-10 K764 | ICD-10: K764 |
| Veno-occlusive disease | ICD-10 K765 | ICD-10: K765 |

^1^ Search for codes in all PMSI, DCIR and CépiDc tables for years 2013 to 2023

ATC: Anatomical, Therapeutic and Chemical Classification, Common Classification of Medical Procedures *[Classification Commune des Actes Médicaux],* CépiDc : Center for Epidemiology of Medical Causes of Death *[Centre d'épidémiologie sur les causes médicales de décès],* CIP: The French national drug identifier for pharmaceutical presentations *[Code Identifiant de Présentation]*, CLD: chronic liver disease, HBV: Hepatitis B virus, HCV: Hepatitis C virus, ICD-10: International Classification of Diseases, 10th revision, DCIR: French outpatient healthcare claims database *[Données de consommations inter régime],* MAFLD: Metabolic dysfunction Associated Liver Disease, MASH: Metabolic dysfunction Associated Steatohepatitis, NABM: Nomenclature of medical biology procedures *[Nomenclature des actes de biologie médicale]*, PCR: Polymerase chain reaction, PMSI: French hospital discharge database *[Programme de médicalisation des systèmes d’information],* UCD*:* smallest pharmaceutical packaging of medicines dispensed at the hospital *[Unités Communes de Dispensation]*
