# Supplementary material for: RESONANCE: Protocol for a Nationwide Cohort Study of Chronic Liver Diseases Using the French National Health Data System
Source: JMIR Res Protoc. 2026 Oct 5;15:e89577. doi: 10.2196/89577 (PMC13680907; doi:10.2196/89577)
Supplement: Supplementary file 4 [file resprot_v15i1e89577_app12.docx]

| Main etiology of CLD (exclusive categories) | Identification Criteria |
| --- | --- |
| Chronic viral hepatitis alone | When a code referring to viral etiology was identified using the specific algorithm (see Multimedia Appendix 11) integrating diagnostic procedures, laboratory tests, and antiviral medication reimbursements between 2013-2023 or in presence of a “viral hepatitis LTC” (even before 2013 for prevalent cases) but no other etiology recorded (alcoholic, metabolic, rare disease) (see Multimedia Appendix 11 for criteria) |
| *Rare-disease related liver disease* (RD) | When rare disease was identified (according to the algorithms on see Multimedia Appendix 11) throughout the follow-up period (2013-2023) or LTC with a rare disease code (excluding E831 and K830) was found (even before 2013 for prevalent cases) but no other etiology recorded (viral, metabolic, alcoholic) (see Multimedia Appendix 11 for criteria). |
| A*lcohol-related liver disease (ALD)* | In case of identification of alcohol disorder or medication (see definitions on see Multimedia Appendix 7) throughout the entire monitoring period (2013-2023) or LTC referring to K70 code (even before 2013 for prevalent cases) but no other etiology recorded (viral, metabolic, rare disease) (see Multimedia Appendix 11 for criteria). |
| Metabolic CLD (MASLD/MASH) | If at least one metabolic risk factors was identified among obesity, type 2 diabetes, sleep apnea, metabolic syndrome (see definitions on see Multimedia Appendix 5) or an ICD-10 code for MASLD (K760) or MASH (K758) throughout the follow-up period (2013-2023) or a LTC for MASLD or MASH (even before 2013 for prevalent cases) but no other etiology recorded (viral, alcoholic, rare disease), (see Multimedia Appendix 11 for criteria). |
| *MASLD with increased alcohol intake (MetALD)* | When metabolic factors (according to the previous definition) were associated to alcohol condition or treatment (according to the previous definition) even if alcohol intake cannot be quantified in the SNDS, but no other etiology recorded (viral, rare disease) |
| *Mixed causes liver disease* (MD) | When two causes or more among viral, alcoholic, rare disease or metabolic were identified according to previous definitions (except association of metabolic and alcoholic classified in MetALD) (see Multimedia Appendix 11 for criteria). |
| U*ndetermined causes of liver disease (UD)* | In the absence of a known cause among viral, rare disease, metabolic or alcohol identified throughout the follow-up period (2013-2023) or a LTC (even before 2013 for prevalent cases) (see Multimedia Appendix 11 for criteria). |

CLD: chronic liver disease, MAFLD: Metabolic dysfunction Associated Liver Disease, MASH: Metabolic dysfunction Associated Steatohepatitis, NAFLD : Non alcoholic fatty liver disease (obsolet term for MAFLD used in previous codage classification) NASH : Non alcoholic steato-hepatitis (obsolet term for MASH used in previous codage classification).
