# Supplementary material for: RESONANCE: Protocol for a Nationwide Cohort Study of Chronic Liver Diseases Using the French National Health Data System
Source: JMIR Res Protoc. 2026 Oct 5;15:e89577. doi: 10.2196/89577 (PMC13680907; doi:10.2196/89577)
Supplement: Supplementary file 5 [file resprot_v15i1e89577_app13.docx]

| Complication | Identification Criteria | Codes^1^ (ICD-10 / ATC / UCD / CCAM) |
| --- | --- | --- |
| Cirrhosis (strict) | Any of the ICD-10 code listed opposite | ICD-10: K700, K702, K703, K717, K74, I85, I982, I983, I864, K704, K720, K721, K767, R18 |
| Portal hypertension | Any of the ICD-10 code listed opposite or transjugulaire intrahepatic portosystemic shunt (TIPS medical procedure) | ICD-10: K766, I85, I982, I983, I864 CCAM: EHCF002 |
| Spontaneous bacterial peritonitis | ICD-10 K65 | ICD-10: K65 |
| Hepatic insuffisance | At least one delivery of rifaximin or ICD-10 code K72 during the entire follow up | ICD-10: K72  ATC: A07AA11, UCD: 9407803 |
| Recurrent or refractory Hepatic encephalopathy | At least one delivery of rifaximin during the entire follow up | ATC: A07AA11, UCD: 9407803 |
| Hepatorenal syndrome | ICD-10 K767 | ICD-10: K767 |
| Ascites | ICD-10 R18 | ICD-10: R18 |
| Alcoholic hepatitis | ICD-10 K701 | ICD-10: K701 |
| Hepatic infarction | ICD-10 K763 | ICD-10: K763 |
| Alcoholic liver failure | ICD-10 K704 | ICD-10: K704 |
| Hyperbilirubinemia | ICD-10 R17 | ICD-10: R17 |
| Decompensation of cirrhosis | Presence of any of the 9 specific complications listed above | Presence of any of the codes from the 9 lines above |
| Cirrhosis (broad) | Any of the conditions listed above | Combination of codes for « cirrhosis (strict) » and those of the 9 following lines (Decompensation of cirrhosis) |
| Liver transplantation | ICD-10 Z944 or procedure related to transplantation | CCAM: HLEA001, HLEA002  ICD-10: Z944 |
| Liver cancer | ICD-10 C22, C220-C229 | ICD-10: C22 |
| HCC | ICD-10 C220 | ICD-10: C220 |
| Cholangiocarcinoma | ICD-10 C221 | ICD-10: C221 |

^1^ Search for codes in all PMSI, DCIR and CépiDc tables for years 2013 to 2023

ATC: Anatomical, Therapeutic and Chemical Classification, CCAM: Common Classification of Medical Procedures *[Classification Commune des Actes Médicaux],* CépiDc : Center for Epidemiology of Medical Causes of Death *[Centre d'épidémiologie sur les causes médicales de décès],* CLD: chronic liver disease, DCIR: French outpatient healthcare claims database *[Données de consommations inter régime],* HCC : Hepatocellular carcinoma, ICD-10: International Classification of Diseases, 10th revision, PMSI: French hospital discharge database *[Programme de médicalisation des systèmes d’information],* TIPS: Transjujular Intra hepatic Porto systemic shunt, UCD*:* smallest pharmaceutical packaging of medicines dispensed at the hospital *[Unités Communes de Dispensation]*
