# Supplementary material for: RESONANCE: Protocol for a Nationwide Cohort Study of Chronic Liver Diseases Using the French National Health Data System
Source: JMIR Res Protoc. 2026 Oct 5;15:e89577. doi: 10.2196/89577 (PMC13680907; doi:10.2196/89577)
Supplement: Supplementary file 6 [file resprot_v15i1e89577_app14.docx]

| Aim | Type | Sub-type | Codes^1^ (ICD-10 / CCAM / ATC) |
| --- | --- | --- | --- |
| Curative treatment | **Surgical resection** |  | CCAM: HLFA002-HLFA020, HLFC002, HLFC003, HLFC004, HLFC027, HLFC032, HLFC037, HLFC801 |
|  | **Radiofrequency tumour destruction (ablation)** |  | CCAM: HLNK001, HLNA007, HLNC003, HLNM001, HLNN900 |
|  | **Liver transplant** |  | CCAM: HLEA001, HLEA002  ICD-10: Z944 |
| Palliative treatment | **Chemoembolization** |  | CCAM: EDLF014, EDLF015, EDLF016, EDLF017, ZZLF900, ZZLF004 |
|  | **Radioembolization** |  | CCAM: EDLL001, EDLL002, ZZNL016 |
|  | **Radiotherapy** |  | ICD-10: Z510 |
|  | **Systemic treatment** | **Targeted therapy** | ATC:  *HCC specific treatments:* Sorafenib: L01EX02, Regorafenib: L01EX05, Cabozantinib: L01EX07, Lenvatinib: L01EX08  *Cholangiocarcinoma specific treatments:* Larotrectinib: L01EX12, Adagrasib: L01XX77, Dabrabenib: L01XE23, Tucatinib: L01EH03, Pemigatinib: L01EN02, Ivisidenib: L01XX62 |
|  |  | **Immunotherapy** | ATC:  *HCC specific treatments:* Atezolizumab: L01FF05, Bevacizumab: L01FG01, Ipilimumab: L01FX04, Nivolumab: L01FF01, Pembrolizumab: L01FF02, Ramucirumab: L01FG02, Durvalumab: L01FF03, Tremelimumab: L01FX20  *Cholangiocarcinoma specific treatments:* Durvalumab: L01FF03, Pembrolizumab: L01FF02, Zanidatamab: L01FD07, Pertuzumab: L01XC13, Trastuzumab: L01XC03 |
|  |  | **Chemotherapy** | ICD-10: Z511 (the specific molecules cannot be identified in the SNDS) |

^1^ Source : codes found in any PMSI, DCIR, CépiDc table for years 2013 to 2023

ATC: Anatomical, Therapeutic and Chemical Classification, CCAM: Common Classification of Medical Procedures *[Classification Commune des Actes Médicaux]*, CépiDc: Center for Epidemiology of Medical Causes of Death *[Centre d'épidémiologie sur les causes médicales de décès],* DCIR: French outpatient healthcare claims database *[Données de consommations inter régime],* ICD-10: International Classification of Diseases, 10th revision, PMSI: French hospital discharge database *[Programme de médicalisation des systèmes d’information],* SNDS: National Health Data System *[Système National des Données de Santé]*
