# Supplementary material for: RESONANCE: Protocol for a Nationwide Cohort Study of Chronic Liver Diseases Using the French National Health Data System
Source: JMIR Res Protoc. 2026 Oct 5;15:e89577. doi: 10.2196/89577 (PMC13680907; doi:10.2196/89577)
Supplement: Supplementary file 7 [file resprot_v15i1e89577_app15.docx]

| **Indicator** | **Basic version** | | **Alternative option** | |
| --- | --- | --- | --- | --- |
|  | **Definition** | **n (%)** | **Definition** | **n (%)** |
| **Hemochromatosis** | ICD-10 code E831 only | 881 (3,3) | Refined algorithm with 2 compulsory conditions : ICD-10 code E831 + ≥ 3 occurrences over 12 months of CCAM code FEJF003 (therapeutic bloodletting) | 536 (2,0) |
| **Primitive sclerosing cholangitis** | ICD-10 code K830 only | 835 (3,1) | Refined algorithm with 3 compulsory conditions :  ICD-10 code K830  +  One ICD-10 codes for chronic inflammatory bowel disease: K50, K51 or M075  **OR**  One CCAM code for gastrointestinal imaging per year for 3 consecutive years : ZCQN002, ZCQJ004, ZCQJ005 or ZCQN001  +  ≥ 3 occurrences of ATC code A05AA02 (ursodeoxycholic acid) | 168 (0,6) |
| **Cirrhosis** | At least one of the ICD-10 codes : K700, K702, K703, K717, K74, I85, I982, I983, I864, K704, K720, K721, K767 or R18. | 11332 (42,5) | At least one of the ICD-10 codes listed opposite or  one of the additional codes relating to complications of cirrhosis :  portal hypertension/varices (I85, I98.2, I98.3, I864, K76.6 or procedure code for transjugular intrahepatic portosystemic shunt),  spontaneous bacterial peritonitis (K65), liver failure (rifaximin treatment, K72, K70,4), hepatorenal syndrome (K76,7), alcoholic hepatitis (K70,1), hepatic infarction (K76,3), hyperbilirubinaemia (R17) | 13488 (50,6) |
| **VHB delta** | ICD-10 code B180 only | 113 (0,42) | ICD-10 code B180 OR ATC code for bulevirtide | 115 (0,43) |
| **MASLD** | At least one of the ICD-10 codes K758 or K760 only | 5500 (20,6) | At least one of the ICD-10 codes K758, K760 OR at least one metabolic risk factors among type 2 diabetes, obesity, metabolic syndrome or sleep apnoea syndrome (as defined by data from the CNAM mapping and/or ICD-10 codes, specific medical procedures or treatments from the PMSI, DCIR and CépiDc tables, see Multimedia Appendix 5), in the absence of codes for chronic infection to HBV/HCV nor rare disease (see Multimedia Appendix 11) nor alcohol (see Multimedia Appendix 7) | 6763 (25,4) |
| **ALD/MetALD** | ICD-10 code K70 only | 6146 (23,1) | At least one ICD-10 code related to a condition associated with alcohol use or a code for the treatment of alcohol dependence (see Multimedia Appendix 7) | 8986 (33,7) |
| **Indicator** | *Definition* | *Mean (SD) / Median [P25-P75]* | *Definition* | *Mean (SD) / Median [P25-P75]* |
| **Charlson score when entering the cohort** | Standard algorithm (Supplementary table 10) | 3,96 (3,2) / 3 [1-6] | Modified version excluding moderate or severe liver disease and liver malignancy | 1,99 (2,6) / 1 [0-3] |
| **Annual GI visits (after entering the cohort, for patients with at least 6-months of follow-up, N=22452)** | All ambulatory visits to GI + all visits at public hospitals | 2,54 (3,18) / 1,67 [0,67-3,38] | All ambulatory visits to GI + all visits at public hospitals if a liver imaging is recorded in the same period | 1,74 (2,6) // 0,89 [0,2-2,25] |

ATC: Anatomical, Therapeutic and Chemical Classification, Common Classification of Medical Procedures *[Classification Commune des Actes Médicaux],* CépiDc : Center for Epidemiology of Medical Causes of Death *[Centre d'épidémiologie sur les causes médicales de décès]*, CLD: chronic liver disease, DCIR: French outpatient healthcare claims database *[Données de consommations inter régimes],* HBV: Hepatitis B virus, HCV: Hepatitis C virus, ICD-10: International Classification of Diseases, 10th revision, DCIR: French outpatient healthcare claims database *[Données de consommations inter régimes],* MASLD: Metabolic dysfunction-Associated Liver Disease, MASH: Metabolic dysfunction-Associated Steatohepatitis, PMSI: French hospital discharge database *[Programme de médicalisation des systèmes d’information]*
