# Supplementary material for: RESONANCE: Protocol for a Nationwide Cohort Study of Chronic Liver Diseases Using the French National Health Data System
Source: JMIR Res Protoc. 2026 Oct 5;15:e89577. doi: 10.2196/89577 (PMC13680907; doi:10.2196/89577)
Supplement: Supplementary file 8 [file resprot_v15i1e89577_app2.docx]

| Code^1^ | Label |
| --- | --- |
| ZCQM001^2^ | Transcutaneous ultrasound of the abdomen, with Doppler ultrasound of digestive vessels |
| ZCQM002^2^ | Transcutaneous ultrasound of the abdomen, with transcutaneous ultrasound of the pelvis and Doppler ultrasound of the digestive vessels |
| ZCQM004^2^ | Transcutaneous ultrasound of the upper abdomen with Doppler ultrasound of the digestive vessels |
| ZCQM005^2^ | Transcutaneous ultrasound of the abdomen, with transcutaneous ultrasound of the pelvis [pelvis] |
| ZCQM006^2^ | Transcutaneous ultrasound of the upper abdomen |
| ZCQM008^2^ | Transcutaneous ultrasound of the abdomen |
| ZCQM010^2^ | Transcutaneous ultrasound of the upper abdomen and pelvis [pelvis] |
| ZCQM011^2^ | Transcutaneous ultrasound of the upper abdomen and pelvis with Doppler ultrasound of the digestive vessels |
| HLQM001^2^ | Transcutaneous ultrasound of the liver and bile ducts |
| HLQM002 | Measurement of liver elasticity by ultrasonic pulse elastography |
| ZCQH001^2^ | CT scan of abdomen and pelvis, with intravenous injection of contrast medium |
| ZCQH002^2^ | CT scan of the abdomen or pelvis, with intravenous injection of contrast medium |
| ZCQK004^2^ | CT scan of abdomen and pelvis, without intravenous injection of contrast medium |
| ZCQK005^2^ | CT scan of abdomen or pelvis, without intravenous injection of contrast medium |
| ZCQJ004^2^ | Remnography [MRI] of the abdomen or pelvis, with intravenous injection of contrast medium |
| ZCQJ005^2^ | Remnography [MRI] of the abdomen and pelvis, with intravenous injection of contrast medium |
| ZCQN001^2^ | Remnography [MRI] of the abdomen and pelvis, without intravenous injection of contrast medium |
| ZCQN002^2^ | Remnography [MRI] of the abdomen or pelvis, without intravenous injection of contrast medium |
| HEQE002 | Oesogastro-duodenal endoscopy |
| HLHB001 | Unguided transcutaneous liver biopsy |
| HLHJ003 | Transcutaneous ultrasound-guided non-targeted liver biopsy |
| HLHJ004 | Transcutaneous ultrasound-guided puncture of liver collections |
| HLHJ005 | Ultrasound-guided transcutaneous liver biopsy of multiple targets |
| HLHJ006 | Transcutaneous ultrasound-guided target liver biopsy |
| HLHH001 | Liver biopsy via the jugular vein with ultrasound and/or X-ray guidance, without intravenous pressure |
| HLHH002 | Transcutaneous CT-guided puncture of liver collections |
| HLHH005 | Jugular liver biopsy with ultrasound and/or X-ray guidance, with intravenous pressure measurement |
| HLHH006 | Transcutaneous CT-guided liver biopsy on a target |
| HLHH007 | Transcutaneous, CT-guided, multi-target liver biopsy |
| HLNK001 | Transcutaneous radiofrequency hepatic tumor destruction with CT guidance |
| HLNA007 | Hepatic tumor destruction with radiofrequency current, by laparotomy |
| HLNC003 | Laparoscopic liver tumor destruction with radiofrequency current |
| HLNM001 | Transcutaneous ultrasound-guided radiofrequency liver tumor destruction |
| HLNN900 | Transcutaneous radiofrequency hepatic tumor destruction with remnographic guidance [MRI] |
| HLFA002 to HLFA020  HLFC002 HLFC003 HLFC004  HLFC027 HLFC032; HLFC037;  HLFC801 | Hepatectomy and liver resection |
| HLEA001 and HLEA002 | Liver collection and transplantation (in addition to ICD-10 code Z94.4) |
| ZZLF900 | Intra-arterial locoregional administration of an anticancer pharmacological agent by an implanted device, without bypass surgery, without hyperthermia |
| ZZLF004 | Locoregional administration of anticancer pharmacological agents via the arterial route, with CEC, with hyperthermia |
| [EDLF014](https://www.aideaucodage.fr/ccam-edlf014) | In situ selective or hyperselective hepatic intraarterial injection of non-radioisotopic anticancer pharmacological agent without particle embolization, by transcutaneous arterial route |
| [EDLF015](https://www.aideaucodage.fr/ccam-edlf015) | Supraselective hepatic in situ intraarterial injection of non-radioisotopic anticancer pharmacological agent without particle embolization via transcutaneous arterial route |
| [EDLF016](https://www.aideaucodage.fr/ccam-edlf016) | Supraselective hepatic in situ intraarterial injection of non-radioisotopic anticancer pharmacological agent with particle embolization via transcutaneous arterial route |
| [EDLF017](https://www.aideaucodage.fr/ccam-edlf017) | In situ selective or hyperselective hepatic intraarterial injection of non-radioisotopic anticancer pharmacological agent with particle embolization, by transcutaneous arterial route |
| ZZNL016 | Internal irradiation by transcutaneous intravenous injection of a radioisotopic pharmacological agent |
| [EDLL001](https://www.aideaucodage.fr/ccam-edll001) | In situ hepatic intraarterial injection of radioisotopic pharmacological agent with particle embolization via the transcutaneous arterial route |
| [EDLL002](https://www.aideaucodage.fr/ccam-edll002) | In situ hepatic intraarterial injection of radioisotopic pharmacological agent without particle embolization, by transcutaneous arterial route |
| ehcf002 | Transjujular Intra hepatic Porto systemic shunt (TIPS) |

Search from years 2013 to 2023 :

1 All the codes are used to construct the proxy for hospital consultations with a GI specialist
2 Codes used to identify surveillance via liver imaging in patients with cirrhosis

CEC: Chemoembolization, CCAM: Common Classification of Medical Procedures *[Classification Commune des Actes Médicaux]*, CT: Computed Tomography, GI: gastro-intestinal, ICD-10: International Classification of Diseases, 10th revision, MRI: Magnetic resonance imaging, TIPS: Transjujular Intra hepatic Porto systemic shunt
