# Supplementary material for: RESONANCE: Protocol for a Nationwide Cohort Study of Chronic Liver Diseases Using the French National Health Data System
Source: JMIR Res Protoc. 2026 Oct 5;15:e89577. doi: 10.2196/89577 (PMC13680907; doi:10.2196/89577)
Supplement: Supplementary file 9 [file resprot_v15i1e89577_app3.docx]

| Code | label | |  |
| --- | --- | --- | --- |
| Usual hepatic blood tests | | | |
| 0516 | Alanine aminotransferase (ALAT, TGP) (Blood) | |  |
| 0517 | Aspartate aminotransferase (ASAT, TGO) (Blood) | |  |
| 1107 | Platelets (thrombocytes): isolated study (NFP) | |  |
| 1104 | Blood count including platelets (NFS, NFP) | |  |
| Fibrosis assessment | |  |  |
| 1000^1^ | Liver fibrosis biological score - fibrotest | |  |
| 1001^1^ | Liver fibrosis biological score - fibrometer V | |  |
| 1002^1^ | Hepatic fibrosis biological score - hepascore | |  |

Search from years 2013 to 2023 :

1 Codes used to construct the proxy for hospital consultations with a GI specialist

ALAT/TGP: Alanine aminotransferase, ASAT, TGO: Aspartate aminotransferase, NABM: Nomenclature of medical biology procedures *[Nomenclature des actes de biologie médicale]*, GI: gastro-intestinal, NFP: platelet count *[Numération formule plaquettaire],* NFS: blood count including platelets *[Numeration Formule Sanguine]*
