# Supplementary material for: RESONANCE: Protocol for a Nationwide Cohort Study of Chronic Liver Diseases Using the French National Health Data System
Source: JMIR Res Protoc. 2026 Oct 5;15:e89577. doi: 10.2196/89577 (PMC13680907; doi:10.2196/89577)
Supplement: Supplementary file 10 [file resprot_v15i1e89577_app4.docx]

a) **Type**

| **Code PS5** | **code B2** | **label** | **Type** |
| --- | --- | --- | --- |
| **1098** | **U03** | CMUc 3 consultation | Consultation |
| **1099** | **U45** | CMUc 4 AND 5 consultation | Consultation |
| **1101** | **APU** | ONE-OFF PUPH consultant opinion | Consultation |
| **1102** | **APY** | OCCASIONAL ADVICE FROM A PSYCHIATRIC CONSULTANT | Consultation |
| **1103** | **APC** | OCCASIONAL ADVICE FROM THE DOCTOR'S CONSULTANT | Consultation |
| **1105** | **CCX** | COMPLEX CONSULTING | Consultation |
| **1109** | **GS** | SPECIALIST CONSULTATION IN GENERAL MEDICINE | Consultation |
| **1110** | **G** | GENERAL MEDICINE CONSULTATION | Consultation |
| **1111** | **C** | C-RATED CONSULTATION | Consultation |
| **1112** | **CS** | CS-RATED CONSULTATION | Consultation |
| **1113** | **CNP** | CNP code CONSULTATION | Consultation |
| **1114** | **CSC** | SPECIFIC CARDIOLOGY CONSULTATION | Consultation |
| **1115** | **CA** | CONSULTATION | Consultation |
| **1117** |  | SPECIALIST CONSULTATION RATED C2 | Consultation |
| **1118** |  | PSYCHIATRIST CONSULTATION RATED C2.5 | Consultation |
| **1140** | **CDE** | SPECIFIC SCREENING CONSULTATION | Consultation |
| **1168** | **CCP** | SEXUAL HEALTH PREVENTION CONSULTATION | Consultation |
| **1922** | **CG** | CONSULTATION | Consultation |
| **2414** | **IC** | ABORTION CONSULTATION | Consultation |
| **2426** | **ICS** | CONSULTATION ABORTION SPECIALIST | Consultation |
| **1056** | **JC** | TELECONSULTATION GENERALIST ABORTION | Teleconsultation |
| **1057** | **JCS** | TELECONSULTATION ABORTION SPECIALIST | Teleconsultation |
| **1096** | **TTE** | TELECONSULTATION BETWEEN ATTENDING PHYSICIAN AND NURSING HOME | Teleconsultation |
| **1157** | **TCP** | Teleconsultation | Teleconsultation |
| **1164** | **TLC** | TELECONSULTATION - LTC AND/OR NURSING HOME | Teleconsultation |
| **1191** | **TC** | ALL-SPECIALTY TELECONSULTATION | Teleconsultation |
| **1192** | **TCG** | GENERALIST TELECONSULTATION | Teleconsultation |
| **3103** | **TLS** | TELECONSULTATION DURING TREATMENT | Teleconsultation |
| **3104** | **TLL** | TELECONSULTATION DEDICATED AREA | Teleconsultation |
| **3105** | **TLD** | HOME TELECONSULTATION | Teleconsultation |

Search from years 2013 to 2023

CMUc: Supplementary Universal Health Coverage *[Couverture Maladie Universelle complémentaire]*, LTC: Long term condition; PUPH: Professor and hospital doctor *[Professeur des Université-Praticien Hospitalier],* CNP: Consultation of Neurology or Psychiatry

b) **Specialties**

| **CODE** | **Speciality** |
| --- | --- |
| 1 | GENERAL MEDICINE |
| 3 | CARDIOVASCULAR PATHOLOGY |
| 8 | GASTRO-ENTEROLOGY AND HEPATOLOGY |
| 9 | INTERNAL MEDICINE |
| 12 | PEDIATRICS (if patient is a minor) |
| 13 | PNEUMOLOGY |
| 22 | SPECIALIST IN GENERAL MEDICINE WITH DIPLOMA |
| 23 | SPECIALTY IN MEDICINE RECOGNIZED BY THE ORDER |
| 31 | PHYSICAL MEDICINE AND REHABILITATION |
| 33 | GENERAL PSYCHIATRY |
| 34 | GERIATRY |
| 35 | NEPHROLOGY |
| 42 | ENDOCRINOLOGY AND METABOLISM |
| 84 | VASCULAR MEDICINE |

Search from years 2013 to 2023
