# Supplementary material for: RESONANCE: Protocol for a Nationwide Cohort Study of Chronic Liver Diseases Using the French National Health Data System
Source: JMIR Res Protoc. 2026 Oct 5;15:e89577. doi: 10.2196/89577 (PMC13680907; doi:10.2196/89577)
Supplement: Supplementary file 11 [file resprot_v15i1e89577_app5.docx]

| Condition | Identification Criteria | ICD-10^1^ Codes | Medical procedure (CCAM codes) or drugs (ATC/CIP Codes) | Exclusion Criteria |
| --- | --- | --- | --- | --- |
| Hypertension^2^ | Presence of any ICD-10 related code OR patients treated with antihypertensive medications, identified via the specific variable available in the CNAM Mapping^3^ | I10, I11, I12, I13 | Specific list of ATC codes with approved indication for hypertension:  C02AB02, C02AC01, C02AC02, C02AC05, C02AC06, C02CA01, C02CA06, C02DC01, C02LA01, C03AA01, C03AA03, C03BA04, C03BA10, C03BA11, C03BX03, C03CA01, C03CA02, C03CA03, C03DA01, C03DB01, C03EA01, C03EA04, C07AA02, C07AA03, C07AA05, C07AA06, C07AA12, C07AA15, C07AA16, C07AA23, C07AB02, C07AB03, C07AB04, C07AB05, C07AB07, C07AB08, C07AB12, C07AG01, C07BA02, C07BB02, C07BB03, C07BB07, C07BB12, C07CA03, C07DA06, C07FB02, C07FB03, C08CA01, C08CA02, C08CA03, C08CA04, C08CA05, C08CA08, C08CA09, C08CA11, C08CA13, C08CX01, C08DA01, C08DB01, C08GA02, C09AA01, C09AA02, C09AA03, C09AA04, C09AA05, C09AA06, C09AA07, C09AA08, C09AA09, C09AA10, C09AA13, C09AA15, C09AA16, C09BA01, C09BA02, C09BA03, C09BA04, C09BA05, C09BA06, C09BA07, C09BA09, C09BA15, C09BB02, C09BB04, C09BB07, C09BB10, C09BX02, C09CA01, C09CA02, C09CA03, C09CA04, C09CA06, C09CA07, C09CA08, C09DA01, C09DA02, C09DA03, C09DA04, C09DA06, C09DA07, C09DA08, C09DB01, C09DB02, C09DB04, C09XA02, C09XA52, C10BX03 | The CNAM mapping variable excludes patients with: - Coronary heart disease - Stroke - Heart failure (acute or chronic) - Peripheral artery disease of lower limbs - Treated end-stage renal disease - Diabetes |
| Dyslipidemia^2^ | Presence of ICD-10 related code OR patients treated with lipid-lowering drugs, identified via the variable available in the CNAM Mapping^4^ | E78 | All medications in ATC class C10 | The CNAM mapping variable excludes patients with: - Coronary heart disease - Stroke - Heart failure (acute or chronic) - Peripheral artery disease of lower limbs - Treated end-stage renal disease - Diabetes |
| Sleep Apnea | Presence of ICD-10 related code | G47.3, R06.3 |  |  |
| Obesity | Presence of ICD-10 related code OR CCAM code | E66 (excluding E66.03, E66.13, E66.83, E66.93) | CCAM: HFCA001, HFCC003, HFFA001, HFFA011, HFFC004, HFFC018, HFGC900, HFKA001, HFKA002, HFKC001, HFMA009, HFMA010, HFMA011, HFMC006–HFMC008, HGCA009, HGCC027 |  |
| Overweight | Presence of ICD-10 related code | E66.03, E66.13, E66.83, E66.93 |  |  |
| Diabetes^2^ | Presence of ICD-10 related code  OR treated patients, identification via the variable available in the CNAM Mapping^5^ | Diabetes : E10–E14  Diabetes complications : G59.0, G63.2, G73.0, G99.0, H28.0, H36.0, I79.2, L97, M14.2, M14.6, N08.3 | *2015-2021:* ATC: A10 (excluding A10BX06 - benfluorex) *2022:* ATC: A10 (excluding A10BX06, non-diabetes GLP-1 analogues, SGLT-2 inhibitors ; but including SGLT-2 inhibitors in combination with METFORMIN: ATC: A10BD15, A10BD20, specific SGLT-2 inhibitors: CIP: 3400927892912 (Jardiance® 25mg), GLP-1 analogues in combination with insulin: ATC: A10AE56, specific GLP-1 analogues: ATC: A10BJ01, A10BJ02, A10BJ05, A10BJ06) | Diagnoses from follow-up care and rehabilitation services are excluded due to risk of false identification |
| Type 2 Diabetes (proxy) | Identified via the diabetes variable as described above, restricting to patients with no ICD-10 code E10 between 2013-2023 |  |  |  |
| Metabolic syndrome (proxy) | **Identified if obesity as well as at least 2 other comorbidities among hypertension, dyslipidemia, type 2 diabetes are identified (according to the definitions above) between 2013-2023** |  |  |  |

*^1^* Search for ICD-10 codes in all PMSI, DCIR and CépiDc tables

*^2^* Specific identification variables for metabolic risk factors in the CNAM mapping database (included in the SNDS) are available for years 2015 to 2022. For years 2013, 2014, and 2023, the identification of the metabolic risk factor solely relied on searching ICD-10 corresponding codes in all PMSI, DCIR, and CépiDc tables. Algorithms for the CNAM mapping variables construction are described in the following report: *CNAM. Méthodologie médicale de la cartographie des pathologies et des dépenses, version G11 (années 2015 à 2022, Tous Régimes). Caisse Nationale d’Assurance Maladie. Direction de la Stratégie, des Etudes et des Statistiques - Département des Etudes sur les Pathologies et les Patients. 2024*.

*^3^* Variables "top_FAntiHTA_med"/"TFC_HTA_EXC" in the CNAM mapping database (see CNAM mapping algorithms category 2.1) identifying people who have received at least 3 deliveries of specific drugs in year n (on different dates), and in the case of large packs at least 1 large and 1 small, or 2 large, but who are not identified as having coronary heart disease or stroke or heart failure - neither acute nor chronic - or obliterative arterial disease of the lower limbs, or treated chronic end-stage renal failure, or diabetes. The list includes all drugs with a reimbursable therapeutic indication for the treatment of hypertension (and their history since January 1, 2000). Antihypertensive drugs with ATC codes beginning with C02, C03, C07, C08, C09, C10 that have no indication, according to their marketing authorization, in the treatment of hypertension are excluded.

*^4^* Variables " top_FHypoLi_med"/"TFC_LIP_EXC" in the CNAM mapping database (see CNAM mapping algorithms category 2.1) identifying people who have received at least 3 deliveries of specific drugs in the year n (on different dates), and in the case of large packs at least 1 large and 1 small, or 2 large, but who are not identified as having coronary heart disease or stroke or heart failure - neither acute nor chronic - or obliterative arterial disease of the lower limbs, or treated chronic end-stage renal failure, or diabetes.

*^5^* Variables "top_FDiabet_ind"/"DIA_CAT_CAT" in the CNAM mapping database (see CNAM mapping algorithms category 3) identifying people who have received at least 3 deliveries of oral or injectable specific antidiabetics or insulin in year n or n-1 (and in the case of large packs at least 1 large and 1 small, or 2 large) on different dates, or people on LTC in year n with ICD-10 codes for diabetes or people hospitalized (in medicine, surgery, obstetrics) in the last 2 years with a ICD-10 diabetes (or diabetes complication) code.

ATC: Anatomical, Therapeutic and Chemical Classification, CCAM: Common Classification of Medical Procedures *[Classification Commune des Actes Médicaux]*, CNAM: National Health Insurance Fund *[Caisse National d’Assurance Maladie],* CépiDc: Center for Epidemiology of Medical Causes of Death *[Centre d'épidémiologie sur les causes médicales de décès],* CIP: The French national drug identifier for pharmaceutical presentations *[Code Identifiant de Présentation]*, DCIR: French outpatient healthcare claims database *[Données de consommations inter régime],* ICD-10: International Classification of Diseases, 10th revision, LTC: long-term condition *[Affection de Longue Durée]* (a legal status in the French healthcare system granting full reimbursement for care related to specific chronic or severe illnesses requiring long-term treatment), PMSI: French hospital discharge database *[Programme de médicalisation des systèmes d’information],* SNDS: National Health Data System *[Système National des Données de Santé]*
