# Supplementary material for: RESONANCE: Protocol for a Nationwide Cohort Study of Chronic Liver Diseases Using the French National Health Data System
Source: JMIR Res Protoc. 2026 Oct 5;15:e89577. doi: 10.2196/89577 (PMC13680907; doi:10.2196/89577)
Supplement: Supplementary file 12 [file resprot_v15i1e89577_app6.docx]

| Identification Method | Description | Codes |
| --- | --- | --- |
| Identification via the variable available in the CNAM Mapping^1^  (top_IRVih_ind/INF_VIH_IND) | LTC associated with HIV-related ICD-10 | LTC N° 7 or associated with  ICD-10: B20, B21, B22, B23, B24, F02.4, Z21 |
|  | Hospitalization for HIV or complication (as main or associated cause) | ICD-10: B20, B21, B22, B23, B24, F02.4, Z21  GHM codes *(2022)*: 25C02, 25M02, 25Z02, 25Z03 |
|  | ≥3 HIV treatment deliveries in a year | ATC: J05AF01, J05AF02, J05AF03, J05AF04, J05AF06, J05AF13, J05AG01, J05AG03, J05AG04, J05AG05, J05AG06, J05AR01, J05AR02, J05AR04, J05AE01, J05AE02, J05AE03, J05AE04, J05AE05, J05AE07, J05AE08, J05AE09, J05AE10, J05AR10, J05AJ01, J05AJ03, J05AJ04 *(2022)*, J05AR06, J05AR08, J05AR09, J05AR13, J05AR18, J05AR19, J05AR20, J05AR21, J05AR24, J05AX07, J05AX08, J05AX09, J05AX12, J05AX23 *(2022)*, J05AX29 *(2022)*, J05AX31 *(2022)* |
|  | HIV-specific biology procedure | NABM: 0805, 0806, 1691, 4117, 4122 |

*^1^* Specific identification variables for HIV in the CNAM mapping database (included in the SNDS) are available for years 2015 to 2022. For years 2013, 2014, and 2023, the identification solely relied on searching ICD-10 corresponding codes in all PMSI, DCIR, and CépiDc tables. Algorithms for the CNAM mapping variables construction are described in the following report: *CNAM. Méthodologie médicale de la cartographie des pathologies et des dépenses, version G11 (années 2015 à 2022, Tous Régimes). Caisse Nationale d’Assurance Maladie. Direction de la Stratégie, des Etudes et des Statistiques - Département des Etudes sur les Pathologies et les Patients. 2024*.

ATC: Anatomical, Therapeutic and Chemical Classification, CNAM: National Health Insurance Fund *[Caisse National d’Assurance Maladie],* CépiDc: Center for Epidemiology of Medical Causes of Death *[Centre d'épidémiologie sur les causes médicales de décès],* DCIR: French outpatient healthcare claims database *[Données de consommations inter régime],* GHM : Diagnosis-Related Groups *[Groupe Homogène de Malades]*, HIV: Human Immunodeficiency Virus, ICD-10: International Classification of Diseases, 10th revision, LTC: long-term condition *[Affection de Longue Durée]* (a legal status in the French healthcare system granting full reimbursement for care related to specific chronic or severe illnesses requiring long-term treatment), NABM: Nomenclature of medical biology procedures *[Nomenclature des actes de biologie médicale]*, PMSI: French hospital discharge database *[Programme de médicalisation des systèmes d’information]*, SNDS: National Health Data System *[Système National des Données de Santé]*
