# Supplementary material for: RESONANCE: Protocol for a Nationwide Cohort Study of Chronic Liver Diseases Using the French National Health Data System
Source: JMIR Res Protoc. 2026 Oct 5;15:e89577. doi: 10.2196/89577 (PMC13680907; doi:10.2196/89577)
Supplement: Supplementary file 13 [file resprot_v15i1e89577_app7.docx]

| Source | Criteria | Codes (ICD-10 / ATC / CIP) |
| --- | --- | --- |
| PMSI DCIR CépiDc | People on LTC, hospitalized or with a cause of death associated to alcohol consumption (clinical diagnoses, alcohol-related conditions, mental/behavioral disorders related to alcohol, etc.) | ICD-10: F10, E244, E512, E52, G312, G621, I426, K292, K70, K700, K701, K702, K703, K704, K709, K860, T510, Z502, Z714, Z721, R780, X45, X65, Y15, Y90, Y91, G721, Y573 |
| PMSI DCIR | Alcohol withdrawal treatment | ATC: N07BB01, N07BB03, N07BB05, N07BB04,  CIP: 3400936743199, 3400892697789, 3400930163528, 3400930163597, 3400930186886, 3400930163665, 3400894495239, 3400894494928, 3400894495000, 3400894495178 |

Search from years 2013 to 2023

ATC: Anatomical, Therapeutic and Chemical Classification, CépiDc: Center for Epidemiology of Medical Causes of Death *[Centre d'épidémiologie sur les causes médicales de décès],* CIP: The French national drug identifier for pharmaceutical presentations *[Code Identifiant de Présentation]*, DCIR: French outpatient healthcare claims database *[Données de consommations inter régime],* ICD-10: International Classification of Diseases, 10th revision, LTC: long-term condition *[Affection de Longue Durée]* (a legal status in the French healthcare system granting full reimbursement for care related to specific chronic or severe illnesses requiring long-term treatment), PMSI: French hospital discharge database *[Programme de médicalisation des systèmes d’information]*
