# Supplementary material for: RESONANCE: Protocol for a Nationwide Cohort Study of Chronic Liver Diseases Using the French National Health Data System
Source: JMIR Res Protoc. 2026 Oct 5;15:e89577. doi: 10.2196/89577 (PMC13680907; doi:10.2196/89577)
Supplement: Supplementary file 14 [file resprot_v15i1e89577_app8.docx]

| Source | Criteria | Codes (ICD-10 / ATC) |
| --- | --- | --- |
| PMSI DCIR CépiDc | People on LTC, hospitalized or with a cause of death associated to mental/behavioral disorders related to tobacco | ICD-10: F17 |
| PMSI DCIR CépiDc | People on LTC, hospitalized or with a cause of death associated to smoking-related conditions | ICD-10: Z72.0, Z71.6, Z508, T65.2 |
| PMSI DCIR CépiDc | People on LTC, hospitalized or with a cause of death associated to respiratory diseases | ICD-10: J41, J42, J43, J44, I73.1 |
| PMSI DCIR | Nicotin substitutes | ATC: N07BA01, N07BA03, N06AX12 |
| PMSI DCIR | Specific respiratory medications | ATC: R03AC18, R03AC19, R03BB04, R03BB05, R03BB06, R03BB07, R03AL04, R03AL05, R03AK04, |

Search from years 2013 to 2023

ATC: Anatomical, Therapeutic and Chemical Classification, CépiDc: Center for Epidemiology of Medical Causes of Death *[Centre d'épidémiologie sur les causes médicales de décès],* DCIR: French outpatient healthcare claims database *[Données de consommations inter régime],* ICD-10: International Classification of Diseases, 10th revision, LTC: long-term condition *[Affection de Longue Durée]* (a legal status in the French healthcare system granting full reimbursement for care related to specific chronic or severe illnesses requiring long-term treatment), PMSI: French hospital discharge database *[Programme de médicalisation des systèmes d’information]*
