# Supplementary material for: RESONANCE: Protocol for a Nationwide Cohort Study of Chronic Liver Diseases Using the French National Health Data System
Source: JMIR Res Protoc. 2026 Oct 5;15:e89577. doi: 10.2196/89577 (PMC13680907; doi:10.2196/89577)
Supplement: Supplementary file 15 [file resprot_v15i1e89577_app9.docx]

| Substance Type | Source^1^ | Criteria Description | ICD-10 Codes |
| --- | --- | --- | --- |
| Cannabis | Variable available in the CNAM Mapping (sup_PAddCan_ind/PSY_ADD_CNB) | ICD-10 codes identifying People on LTC or hospitalized for cannabis-related mental/behavioral disorders | ICD-10: F12 |
| Other Drugs | Variable available in the CNAM Mapping (sup_PAddAut_ind/PSY_ADD_AUT) | ICD-10 codes identifying People on LTC or hospitalized for mental/behavioral disorders related to drugs other than alcohol, tobacco, cannabis | ICD-10: F11, F13, F14, F15, F16, F18, F19 |

^1^ Specific identification variables for drug use in the CNAM mapping database (included in the SNDS) are available for years 2015 to 2022 only (no other search was performed for the remaining years). Algorithms for the CNAM mapping variables construction are described in the following report: *CNAM. Méthodologie médicale de la cartographie des pathologies et des dépenses, version G11 (années 2015 à 2022, Tous Régimes). Caisse Nationale d’Assurance Maladie. Direction de la Stratégie, des Etudes et des Statistiques - Département des Etudes sur les Pathologies et les Patients. 2024*.

CNAM: National Health Insurance Fund *[Caisse National d’Assurance Maladie],* ICD-10: International Classification of Diseases, 10th revision, LTC: long-term condition *[Affection de Longue Durée]* (a legal status in the French healthcare system granting full reimbursement for care related to specific chronic or severe illnesses requiring long-term treatment), SNDS: National Health Data System *[Système National des Données de Santé]*
